# Inducing Propeller Chirality in Triaryl Boranes with Chiral Amines

**DOI:** 10.1002/chem.202202812

**Published:** 2022-10-25

**Authors:** Michael Kemper, Sven Reese, Elric Engelage, Christian Merten

**Affiliations:** ^1^ Ruhr Universität Bochum, Organische Chemie II Universitätsstrasse 150 44801 Bochum Germany

**Keywords:** chirality, circular dichroism, stereochemistry, supramolecular chemistry, vibrational spectroscopy

## Abstract

Stabilization of chiral propeller conformations in triaryl compounds is challenging due to generally low racemization barriers. Nonetheless, it was recently found that chiral conformational preferences can be induced to triaryl boranes by incorporating point‐chiral alkylether chains to the aryl blades and subsequently locking the structure with ammonia. A four‐point interaction, meaning that the cooperative effects of Lewis‐adduct formation and three hydrogen bonds, was proposed as stabilizing mechanism. Herein, it was shown that three such strong interactions suffice to introduce a preferential propeller handedness. Although DFT calculations predict no noteworthy preferences for either P‐ or M‐chiral propellers for some of the investigated triarylborane–amine adducts that were prepared with chiral primary amines, vibrational circular dichroism (VCD) spectroscopic characterizations revealed that there is indeed a measurable excess of one propeller handedness. Furthermore, the steric demand of the amine was found to play a key role in the induction process and especially in preventing blade rotations.

## Graphical abstract

**A three‐point interaction** between a chiral amine and a triarylborane is shown to suffice to induce a preferential propeller handedness to the borane. Rotation of aryl blades can be avoided by incorporation of naphthyl instead of phenyl and a bulky amine component.

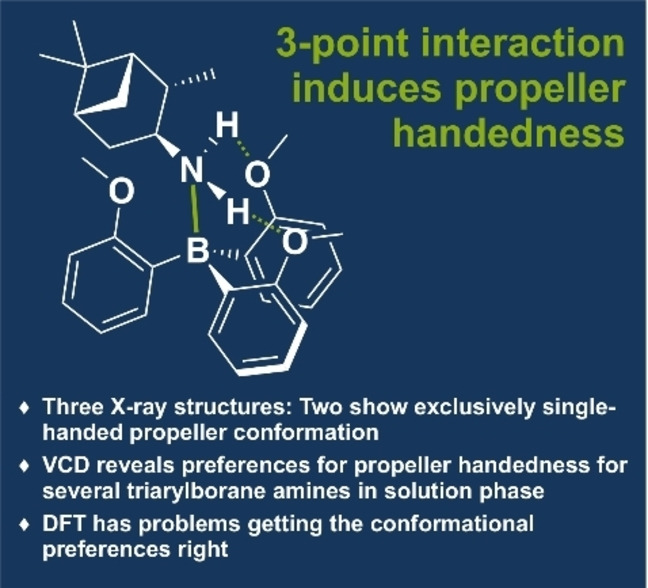

## Introduction

Molecular propellers keep attracting the interest of (stereo‐)chemists^[1]^ since Mislow's seminal papers on the conformation of triaryl compounds.^[2]^ The preparation of molecular propellers with different pivot atoms^[3]^ and of various spatial dimensions has stimulated many applications in supramolecular chemistry and molecular recognition.^[4]^ Interestingly, although already discussed by Mislow as a model system, it was only recently, that Ito and co‐workers realized the first enantiopure propeller‐shaped triaryl.^[5]^ While triaryl boranes indeed exist in enantiomeric propeller conformations in solid state,^[6]^ isolation in solution is usually prevented by rapid exchange between the two enantiomeric conformers. Hence, the chiral propeller conformation had to be stabilized by large 1,3‐diethynylphenylenes as aryl substituents, which effectively interlock the blades and thus prevented racemization.

**Scheme 1:**
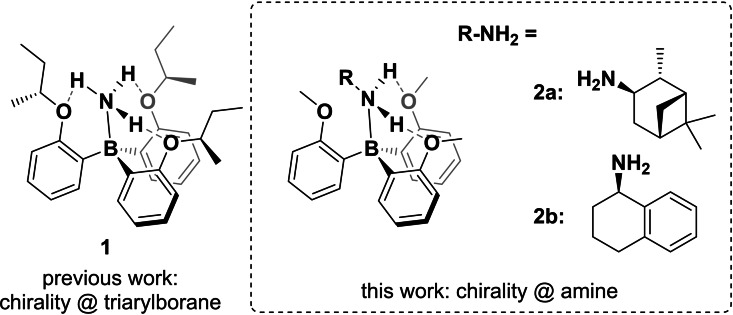
Chiral triarylborane ammonia complex stabilized by a four‐point interaction introduced in previous work and the target molecule of this study featuring the chiral information in the primary amine substituents.

Coordination of the central boron with an amine leads to a tetrahedral boron center^[7]^ and the formation of chiral, yet racemic propeller structures. Similar to boranes, isolation of enantiopure B−N adducts with predominant handedness of the borane propeller are prevented by low racemization barriers. In order to increase the racemization barrier, we followed up on work by Trauner et al.^[8]^ and recently introduced a series of novel chiral triarylborane ammonia complexes such as **1** (cf. Scheme 1).^[9]^ They utilize a four‐point interaction comprising of the interaction through the B−N bond and additional three hydrogen bonds between NH_3_ and ether oxygens on the three aryl blades to stabilize a preferential propeller chirality. While we did not find such preference in solid state, vibrational circular dichroism (VCD)^[10]^ and electronic circular dichroism (ECD)^[11]^ spectroscopies were used to confirm these preferences in solution phase. An increased distance of the stereogenic centers in the ether side groups from the propeller pivot point was further found to lead to a drop in stereochemical induction. The theoretical analyses of the experimental VCD spectra were complicated by the fact that the computationally predicted conformational preferences did not match with the experimental observations: A weak intramolecular C^α^−H⋅⋅π interaction between each of the stereocenters in the ether side groups and the adjacent aryl blades, which was only present in one of the propeller conformations, led to an artificial stabilization of these conformers. In solution phase, however, these C^α^−H⋅⋅π interactions were thought to be cleaved by solute‐solvent interactions.

Based on these results, we became interested in elucidating whether moving the stereoinformation into the amine‐component would still give rise to induced propeller chirality in the triaryl borane. However, incorporating stereoinformation via the amine necessarily leads to a loss of one of the stabilizing hydrogen bonds. Consequently, an effective stereoinduction was thus anticipated to require sterically demanding amines, but at the same time, steric interactions may also be found to prevent formation of the central amine‐borane B−N bond.

## Results and Discussion

**Scheme 2:**
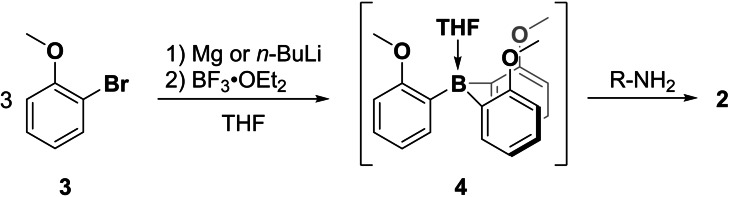
Preparation of triarylborane‐amines **2**.

We prepared the borane amine **2** **a** following the same route as for **1** (Scheme 2).^[9]^ In brief, formation of the Grignard‐reagent of *ortho*‐bromo phenyl methyl ether **3** or, alternatively, *ortho*‐lithiation of **3** with *n*‐butyl lithium, and subsequent reaction with trifluoroborate etherate led to the in situ formation of intermediate **4**. The target compound **2** **a** was subsequently obtained as white solid by complexation through quenching of the reaction mixture with the enantiopure isopinocampheyl amine.

**Figure 1:**
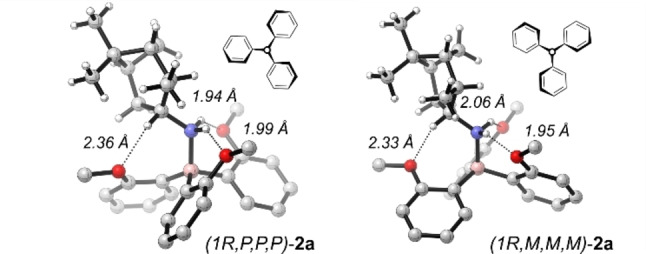
Molecular structures of *(1R,P,P,P)*‐ and *(1R,M,M,M)*‐**2** **a** obtained from X‐ray crystallography. Note that both P‐ and M‐propeller conformations are found in the same unit cell. Further crystallographic information can be found in the Supporting Information file. A P‐orientation of a blade is defined by a negative N−B⋅⋅C−O torsional angle, while an M‐orientation is given by a positive N−B⋅⋅C−O angle.

Crystals of **2** **a** suitable for X‐ray analysis were obtained from hexane (cf. Figure 1). The analysis showed both propeller conformations in the same unit cell. Furthermore, in both propeller structures, all three methoxy groups, even the one that is not in an intramolecular N−H⋅⋅O hydrogen bond, point towards the amine. In light of the problematic role that C^α^−H⋅⋅π interactions played in the analysis of the preferred conformations of **1**, it is worth noting that a C−H⋅⋅O interaction may be postulated for both propeller structures. It should be stressed again that the presence of both propellers in the solid state does not mean that the chiral amine is not inducing a detectable preference for either propeller structure in solution phase. In order to further investigate the conformational preferences of **2** **a** we carried out a VCD spectroscopic analysis.

We first carried out a comprehensive conformational analysis of (1*R*)‐**2** **a** at the B3LYP/6‐31+G(2d,p)/ IEFPCM(CHCl_3_) level of theory to later enable the analysis of the experimental IR and VCD spectra by comparison to the computed spectra.^[10d]^ Due to the rigid structure of the amine, we only had to evaluate the conformations of the triaryl borane moiety: it can adopt either a complete propeller conformation, denoted PPP and MMM, or conformations in which one aryl blade is aligned parallel to the B−N bond, denoted PP0 and MM0. In addition, we considered structures in which one or more aryl blades have the methoxy substituents pointing away from the amine (denoted *n*u for structures with *n* methoxy groups pointing towards the amine). A total of 18 conformers were found for **2** **a**, but the major conformational weight (>95 %) lied on the **2** **a**‐PPP^3u^ and **2** **a**‐MMM^3u^ conformers, which were also found in the solid‐state structure. Interestingly, the computations also did not predict any significant preference for either structure, with an energy difference of almost zero.

**Figure 2:**
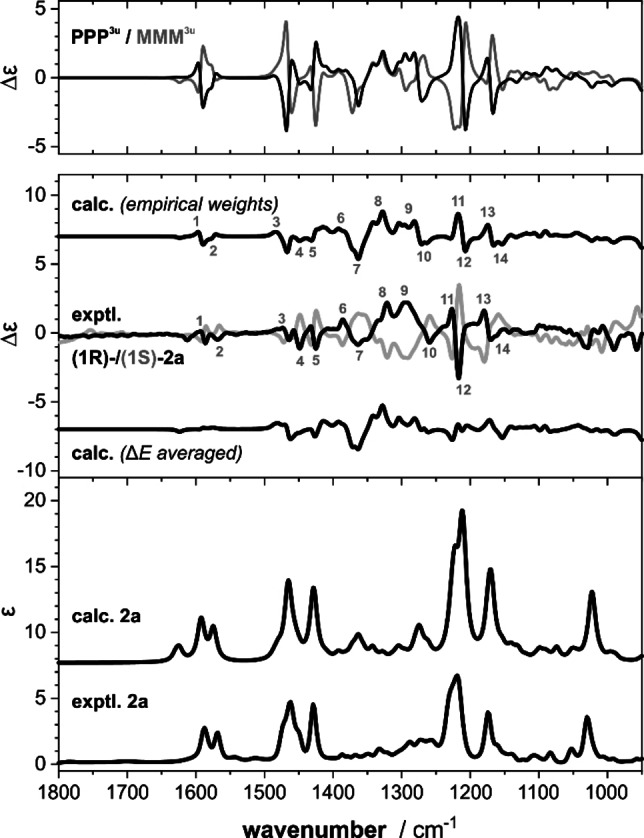
Experimental IR and VCD spectra of **2** **a** (82 mM, 100 μm path length, CDCl_3_) compared to computed spectra of (1*R*)‐**2** **a** obtained by Boltzmann‐averaging over all conformers (ΔE_ZPC_ weighted) and after manually adjusting the contributions of **2** **a**‐PPP^3u^ and **2** **a**‐MMM^3u^ to 60 : 40 (empirical weights). The top panel shows the single conformer VCD spectra of **2** **a**‐PPP^3u^ and **2** **a**‐MMM^3u^. Numbers indicate band assignments. Molar absorptivity ϵ and differential molar absorptivity Δϵ are given in units of 10^2^ M^−1^ cm^−1^ and 10^−2^ M^−1^ cm^−1^.

The experimental IR and VCD spectra of **2** **a** are compared to the predicted ones in Figure 2. While most experimental features were well reproduced, the experimentally observed strong couplet arising from the C−O stretching vibrations (experimental bands 11/12) of the methoxy groups is immediately noted as missing or at least as much too weak in the spectra simulated based on the relative zero‐point corrected energies (ΔE_ZPC_). We thus had a closer look at the single conformer spectra of the PPP^3u^ and MMM^3u^ conformers of **2** **a** in order to identify potentially characteristic signatures. While the IR spectra of the two conformers were virtually identical, the two VCD spectra showed mirror image relation over most of the investigated fingerprint region (cf. Figure 2, top VCD panel). A minor deviation from the computed PPP^3u^/MMM^3u^ ratio of ∼50 : 50 towards 60 : 40 (denoted empirical weights in Figure 2) generated the correct spectral features in the range of the C−O stretching modes (bands 11/12, 1250–1200 cm^−1^) and also improved the signature of the C=C stretching modes in the range 1625–1550 cm^−1^ (bands 1/2). The VCD spectroscopic analysis thus confirms that a preferential propeller chirality can be induced by a chiral amine as source of stereochemical information.

As a further confirmation, we also recorded the ECD spectrum of **2** **a**. It features only one strong couplet with bands of opposite sign at 205 and 193 nm (cf. Fig S1), and according to the spectra simulations, the two PPP^3u^/MMM^3u^ weights differ only in the intensity of these two bands. Thus, a slight preference towards P‐structures could be proposed, however a clear conclusion can only be drawn from the VCD analysis.

**Figure 3:**
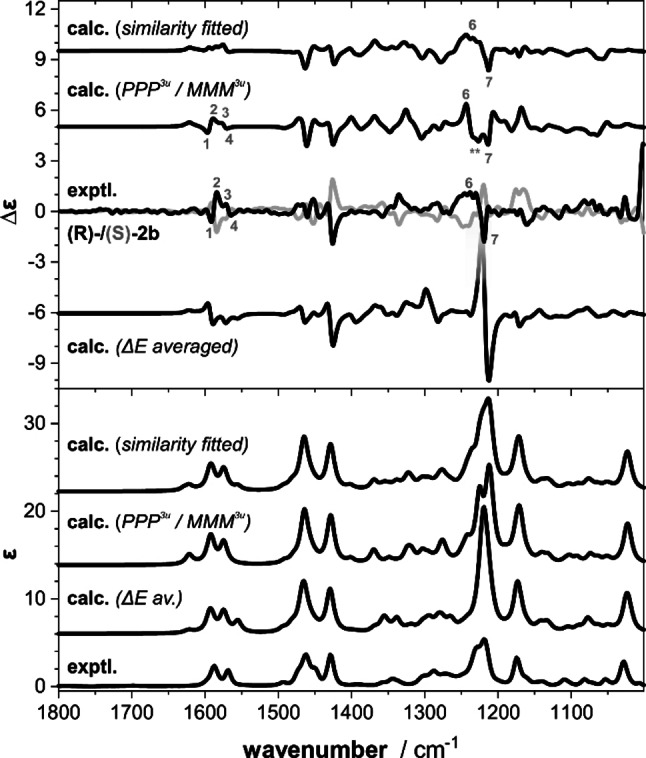
Experimental IR and VCD spectra of **2** **b** (0.135 M, 100 μm path length, DCM‐d_2_) compared to computed spectra of (*R*)‐**2** **b** obtained by Boltzmann‐averaging over all conformers (ΔE_ZPC_ weighted), after manually adjusting the contributions of **2** **b**‐PPP^3u^ and **2** **b**‐MMM^3u^ (empirical weights) and by fitting based on highest similarity. Numbers indicate band assignments. Molar absorptivity ϵ and differential molar absorptivity Δϵ are given in units of 10^2^ M^−1^ cm^−1^ and 10^−2^ M^−1^ cm^−1^.

Assuming that a sterically more demanding amine with aromatic groups may be better suited to induce a strong chiral conformational preference to the triaryl propeller, we prepared **2** **b** following the route outlined in Scheme 2. Unfortunately, we did not obtain crystals suitable for X‐ray analysis, but the experimental IR and VCD spectra of **2** **b** (cf. Figure 3) showed again a very good mirror‐image relation. The computational analysis of **2** **b** gave three highly populated conformers, all featuring the three methoxy groups pointing towards the amine. With conformer weights of 69 % for **2** **b**‐PP0^3u^, 15 % for **2** **b**‐PPP^3u^ and 14 % for **2** **b**‐MMM^3u^, the computed conformational distribution is clearly in favor of P‐chiral propeller conformations (cf. Supporting Information for more details). However, the comparison of the experimental IR and VCD spectra with those simulated for this conformational distribution reveals a clear mismatch of several signatures. In particular the two aforementioned regions of the C=C and C−O stretching modes (bands 1–4 and 6/7, 1625–1550 and 1250–1200 cm^−1^) of the VCD spectra are not well reproduced by the simulation, with the first range actually being predicted with basically opposite signs.

As demonstrated for **2** **a** in Figure 2, the single conformer spectra of **2** **b** (cf. Figure S2) and in particular the signature of the VCD bands 1–4 is characteristic for the propeller chirality (+/− for P, −/+ for M). An inversion of the population, that means, shifting the conformational preferences to about 65 % of the **2** **b**‐MMM^3u^‐propeller conformer, thus led to an improved match in the C=C stretching mode region. In fact, using only the **2** **b**‐PPP^3u^ and **2** **b**‐MMM^3u^ conformations, the VCD pattern in the range 1625–1550 cm^−1^ (bands 1–4) could be almost exactly reproduced. Nonetheless, a mismatch in the C−O stretching region, that is an additional negative feature marked with double asterisk in Figure 3, indicated that considering only two conformers constituted a too simplified model. The broad appearance of band 6 further supported the assumption that other conformers must contribute as well.

Attempting to find a conformational distribution that gives a better resemblance of the experimental spectra, we evaluated thousands of linear combinations of the eight optimized conformers of **2** **b** by means of an automated similarity analysis. This procedure yielded many combinations with very high similarity measures (i. e. large overlap integrals; Figure S3); characteristic to all of them was an excess of M‐propeller conformers and contributions of the **2** **b**‐MMM^2u^ conformer. Figure 3 shows a selected spectrum, from which it can immediately be seen that the match with the experimental signatures could indeed be strongly improved. Hence, while the exact conformational preferences are obviously difficult to extract from the VCD signatures, the preference for M‐propeller conformations could unambiguously be deduced.

**Figure 4:**
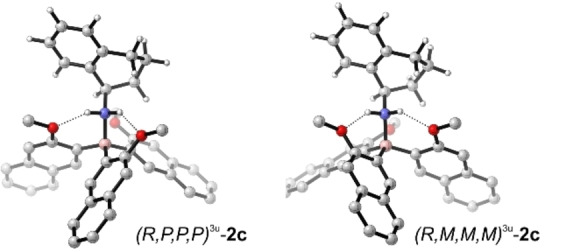
Optimized structures of **2** **c**‐PPP^3u^ and **2** **c**‐MMM^3u^.

**Figure 5:**
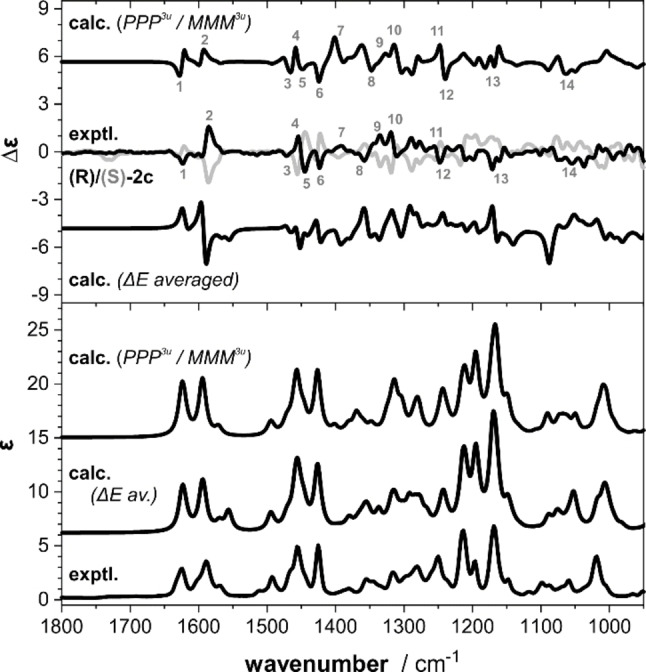
Experimental IR and VCD spectra of **2** **c** (0.135 M, 100 μm path length, DCM‐d_2_) compared to computed spectra of (*R*)‐**2** **c** obtained by Boltzmann‐averaging over all conformers (ΔE_ZPC_ weighted) and after manually adjusting the contributions of **2** **c**‐PPP^3u^ and **2** **c**‐MMM^3u^ (empirical weights). Numbers indicate band assignments. Molar absorptivity ϵ and differential molar absorptivity Δϵ are given in units of 10^2^ M^−1^ cm^−1^ and 10^−2^ M^−1^ cm^−1^.

The optimized conformational distribution of **2** **b** giving the well‐matching similarity fitted spectra shown in Figure 3 had significant contributions of conformers with the methoxy group pointing away from the amine (2u‐structures with either M‐ or P‐blades contribute together with about 45 %). Hypothesizing that changing the phenyl to naphthyl blades would introduce enough steric hindrance to avoid such blade rotations, we prepared the trinaphthyl borane **2** **c** (Figure 4). The computational analysis revealed a similar conformational distribution as for **2** **b** despite the extended blade size, but no notable increase in energy of 2u‐structures. As found for the previous two examples, the predicted VCD pattern in the C=C stretching region again did not match with the experimental spectrum (Figure 5), suggesting that the conformational preferences may once more be different from the computed ones. Likewise, putting more weight on the MMM^3u^ conformer (70 %; 30 % PPP^3u^) gave a much better match with the experimental signatures: not only does the pattern of bands 1/2 and in the range 3–7 improve notably, also the signatures 11–14 match much better with the experiment.

**Scheme 3:**
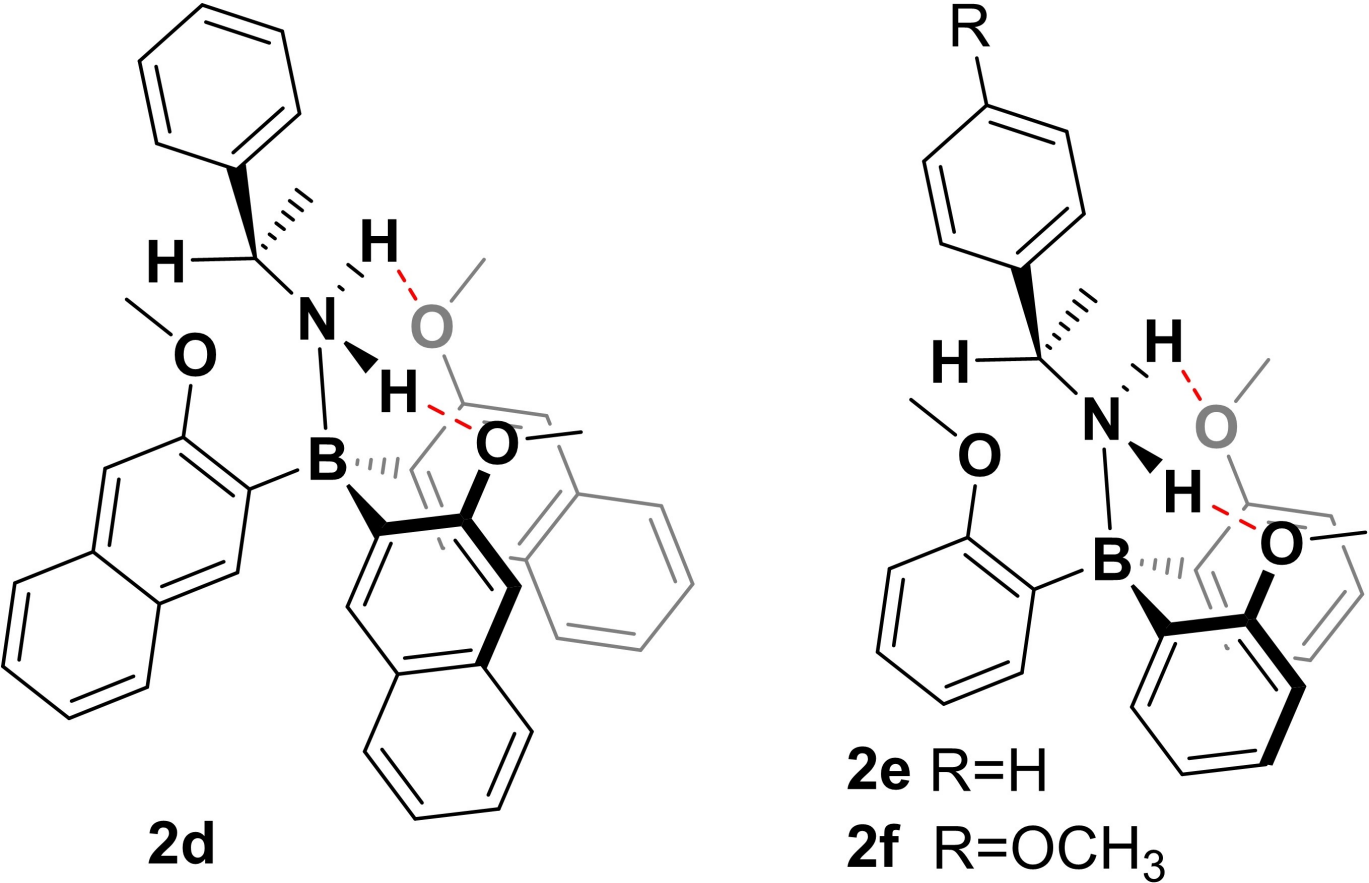
Structures of triarylborane amines **2** **d**–**f** derived from α‐methylbenzyl amine.

Finally, we decided to reduce the size of the amine and incorporated α‐methylbenzyl amine (αMBA) and p‐methoxy‐αMBA giving **2** **d**–**f** (cf. Scheme 3). The VCD spectra of all three compounds are shown in the Supporting Information file and we herein only briefly note that the theoretical analysis of the conformational preferences became increasingly complicated. For the trinaphthylborane amine **2** **d**, neither the Boltzmann‐averaged VCD spectrum nor a spectrum generated based on only the **2** **d**‐MMM^3u^ and ‐PPP^3u^ conformers could explain the experimentally observed spectra (Figure S4). Using our similarity fitting tool, which we successfully utilized for the analysis of **2** **b** as well, we found a reasonable match that included about 50 % of only the **2** **d**‐MMM^2u^, so a propeller with one rotated blade. However, we did not reach the overall quality of match as seen for **2** **b**.

Crystals suitable for X‐ray analysis could be obtained for both **2** **e** and **2** **f** (cf. Supporting Information for more details). Interestingly, in both cases, a single propeller handedness was observed in the solid state. The propeller structures of **2** **e** and **2** **f** nonetheless differ, as **2** **e** features a PPP^2u^ conformation, while a PPP^3u^ was found for **2** **f**. The experimental IR and VCD spectra of **2** **e** and **2** **f** are reported in the Supporting Information file (Figure S5 and S6). Despite the experiences gathered with **2** **a**–**d**, we did not succeed in finding a convincing agreement with computed spectra. Especially for **2** **e**, we attribute this to an extremely flat potential energy landscape of the amine, which allows for small conformational changes at basically no energetic costs. It is thus likely that large amplitude motions of the floppy amine render the spectra complicated to understand.

## Conclusion

Summarizing our study on the triarylborane amines **2** **a**–**f**, we have shown that the chiral amine component indeed induces a preferential propeller handedness. Although our DFT calculations failed to correctly predict these preferences, characteristic VCD spectral patterns enabled us to unambiguously confirm it to range between about 60 : 40 to 70 : 30 for **2** **a**–**d**. This finding is in line with our previous results on **1** and its derivatives,^[9]^ which carry the chiral information in the ether side chains. The approximated conformer ratio translates into an energy difference of only about 0.25 to 0.5 kcal mol^−1^ (assuming a two‐state model). It may be claimed that this is well within the error of DFT‐predicted energies. However, based on our experience in using VCD spectroscopy to characterize conformational preferences of chiral molecules in solution phase,^[12]^ we rather assume that this change in the conformational distribution is due to (weak) solute‐solvent interactions not included in the calculations. Considering that C^α^−H⋅⋅O interaction between the amines’ stereocenters and the ether oxygen of the dangling aryl blade, which were found in the PPP^3u^ and MMM^3u^ structures, could easily be broken by solvent molecules that each face of the aryl blades could act as a weak C−H hydrogen bond acceptor, and that each dangling blade could rotate to favor interactions between the solvent and the ether group, it seems very likely that subtle solute‐solvent interactions affect the equilibrium. Although these influences are difficult to capture in the calculations, the fact that VCD spectroscopy can still resolve the conformational preferences makes it the ideal method to further explore the use of these triarylborane amines in applications, such as chiral recognition or as supramolecular building block.

## Experimental Section

**Synthesis of tris‐(*o‐*methoxyphenyl)‐borane isopinocampheylamine complex (2** **a)**: In a heat dried three‐neck flask, (315.9 mg, 13.0 mmol, 4 equiv.) were suspended in THF_abs_ (10 mL) under argon atmosphere. 2‐bromoanisol (1.8 g, 1.2 mL, 9.78 mmol, 3 equiv.) was slowly added upon which the solvent started to reflux gently. Additional THF (10 mL) was added and the mixture was refluxed for 6 h. The mixture was cooled down to 0 °C and BF_3_⋅Et_2_O (462.7 mg, 0.4 mL, 3.26 mmol, 1 equiv.) was added. The mixture was stirred for additional 16 h at room temperature and subsequently quenched with enantiopure isopinocampheylamine (500.0 mg, 0.6 mL, 3.26 mmol, 1 equiv.). The resulting white precipitate was re‐dissolved by addition of EtOAc (30 mL) and the organic phase was washed with water (3×10 mL). The organic phase was separated and dried with anhydrous sodium sulfate. Removal of the solvent gave a yellowish viscous oil, which was purified by crystallization of the product from ethanol giving a colorless, crystalline solid. Yield: 159.7 mg, 0.33 mol, 10 %. ^1^H NMR (300 MHz, ACN‐*d_3_*): δ/ppm=7.13–7.08 (3H, m, C*H*‐ar), 6.89‐6.84 (6H, m, C*H*‐ar), 6.73‐6.68 (3H, m, C*H*‐ar), 6.61 (1H, m, N*H*), 6.25 (1H, m, N*H*), 3.67 (9H, *s*, O‐C*H*_3_), 3.37‐3.27 (1H, m, C*H*), 2.41‐2.34 (1H, m, C*H*), 2.05‐1.98 (1H, m, C*H*), 1.85‐1.66 (4H, m, C*H*), 1.14 (3H, s, C*H*_3_), 0.97 (1H, d, ^3^J_H−H_=9.7 Hz, C*H*), 0.94 (3H, d, ^3^J_H−H_=7.2 Hz, C*H*_3_), 0.70 (3H, s, C*H*_3_). ^13^C NMR (75 MHz, ACN‐*d_3_*): δ/ppm=163.54, 137.52, 127.46, 120.90, 110.82, 55.24, 52.97, 48.70, 39.03, 34.96, 33.57, 28.04, 23.30, 20.38. ^**11**^ **B** **NMR** (128 MHz, ACN‐d_*3*_): δ/ppm=−2.27.

**Synthesis of tris‐(*o‐*methoxyphenyl)‐borane tetrahydronaphthyl amine complex (2** **b)**: Following the procedure described for **2** **a**, the complex **2** **b** was prepared from 2‐bromoanisol (3.76 g, 2.49 mL, 20.1 mmol, 3.0 equiv.), magnesium turnings (505 mg, 20.8 mmol, 3.1 equiv.), BF3⋅Et_2_O (951 mg, 0.84 mL, 6.7 mmol, 1 equiv.) and (R)/(S)‐tetrahydronaphthylamine (986 mg, 0.98 mL, 6.7 mmol, 1 equiv.). Washing of the crude product with methanol afforded (R)‐**2b** and (S)‐**2b** as a colorless solid. Yield: 165.6 mg, 0.35 mmol, 5 %. ^1^H NMR (300 MHz, ACN‐*d_3_*): δ/ppm=7.24–7.13 (6H, m, C*H*‐ar), 6.93–6.90 (4H, m, C*H*‐ar), 6.82–6.72 (7H, m, C*H*‐ar, N*H*), 6.66 (1H, m, N*H*), 4.50–4.46 (1H, m, C*H*), 3.62 (9H, s, O‐C*H*_3_), 2.88–2.79 (1H, m, C*H*_2_), 2.72–2.62 (1H, m, C*H*_2_), 2.15 (1H, s, C*H*_2_), 1.94–1.79 (2H, m, C*H*_2_), 1.76–1.65 (2H, m, C*H*_2_). ^13^C NMR (75 MHz, ACN‐*d_3_*): δ/ppm=163.65, 138.71, 138.52, 137.82, 130.31, 129.68, 128.51, 127.83, 127.18, 121.05, 110.86, 55.18, 51.34, 29.86, 28.03, 19.44. ^11^B NMR (128 MHz, ACN‐*d_3_*): δ/ppm=‐1.48.

**Synthesis of tris‐(2** **methoxy‐3‐naphthyl)‐borane tetrahydronaphthyl amine complex (2** **c)**: In a heat dried schlenk flask, the 2‐methoxynaphthalene (5.0 g, 31.6 mmol, 3.0 equiv.) was dissolved in THF_abs_ (5 ml) under argon atmosphere. The solution was cooled to 0 °C and a solution of *n*‐butyl lithium (23.7 mL, 1.6 M in hexanes, 38.0 mmol, 3.8 equiv.) was added dropwise upon which the solution turned dark red. After complete addition, the mixture was allowed to warm to room temperature and was stirred for 1 h at that temperature. Subsequently, the mixture was again cooled to 0 °C before BF_3_⋅Et_2_O (1.48 g, 1.29 mL, 10.4 mmol, 1.0 equiv.) was added dropwise upon which the reaction mixture turned black. The mixture was stirred an additional 16 h at room temperature after which the mixture showed a bright orange color. Quenching with enantiopure tetrahydronaphthylamine (1.53 g, 1.51 mL, 10.4 mmol, 1.0 equiv.) afforded a white precipitate, which was re‐dissolved by adding EtOAc (30 mL) and the solution was washed with water (3×10 mL). The organic phase was separated and dried with anhydrous sodium sulfate. Removal of the solvent gave a yellowish viscous oil, which was purified by column chromatography on silica (eluent: *n*−Hexane/EtOAc=30/1 to 10/1) giving **2c** as colorless solid. Yield: 1.11 g, 1.8 mmol, 18 %. ^1^H NMR (300 MHz, DCM‐*d_2_*): δ/ppm=7.78–7.75 (3H, m, C*H*‐ar), 7.49–7.46 (3H, m, C*H*‐ar), 7.38–7.33 (6H, m, C*H*‐ar), 7.23–7.11 (9H, m, C*H*‐ar), 7.04–7.01 (1H, m, C*H*‐ar), 6.94–6.90 (1H, m, N*H*), 6.74–6.67 (1H, m, N*H*), 4.73–4.69 (1H, m, C*H*), 3.77 (9H, *s*, O‐C*H*_3_), 2.88–2.81 (1H, m, *CH*_2_), 2.74–2.64 (1H, m, *CH*_2_), 2.04–1.99 (1H, m, *CH*_2_), 1.86–1.70 (3H, m, *CH*_2_). ^13^C NMR (75 MHz, DCM‐*d_2_*): δ/ppm=162.06, 137.67, 137.47, 136.92, 133.94, 129.58, 129.32, 127.67, 127.64, 126.26, 125.82, 124.98, 122.67, 104.19, 54.86, 53.96, 53.60, 53.24, 50.83, 29.20, 27.12, 18.53. ^11^B NMR (128 MHz, DCM‐*d_2_*): δ/ppm=−1.29.

**Synthesis of tris‐(2‐methoxy‐3‐naphthyl)‐borane α‐methylbenzyl amine complex (2** **d)**: Following the procedure described for **2** **c**, compound **2** **d** was prepared from 2‐methoxynaphthalene (5.0 g, 31.6 mmol, 3.0 equiv.), *n*‐butyllithium (23.7 mL, 1.6 M in hexanes, 38.0 mmol, 3.8 equiv.), BF_3_⋅Et_2_O (1.48 g, 1.29 mL, 10.4 mmol, 1.0 equiv.) and enantiopure α‐methylbenzylamine (1.26 g, 1.34 mL, 10.4 mmol, 1.0 equiv.). The crude product was purified by column chromatography on silica (eluent: *n*−Hexane/EtOAc=30/1 to 10/1) to afford **2** **d** as a colorless solid. Yield: 1.56 g, 2.5 mmol, 24 %. ^1^H NMR (300 MHz, DCM‐*d_2_*): δ/ppm=7.72–7.69 (3H, m, C*H*‐ar), 7.58 (3H, s, C*H*‐ar), 7.52–7.49 (3H, m, C*H*‐ar), 7.40–7.28 (6H, m, C*H*‐ar), 7.28–7.16 (5H, m, C*H*‐ar), 7.11 (1H, s, C*H*‐ar), 6.74‐6.71 (1H, m, N*H*), 6.62‐6.60 (1H, m, N*H*), 4.35‐4.26 (1H, m, C*H*), 3.67 (9H, *s*, O‐C*H*_3_), 1.26 (3H, d, ^3^J_H−H_=6.7 Hz, C*H*_3_). ^13^C NMR (75 MHz, DCM‐*d_2_*): δ/ppm=162.32, 145.25, 136.48, 134.13, 129.86, 129.39, 128.10, 127.97, 126.31, 126.25, 125.29, 123.09, 104.51, 54.95, 54.40, 54.84, 53.68, 22.32. ^11^B NMR (128 MHz, DCM‐*d_2_*): δ/ppm=−1.64.

**Synthesis of tris‐(*o‐*methoxyphenyl)‐borane α‐methylbenzyl amine complex (2** **e)**: Following the procedure reported for **2** **a**, compound **2** **e** was prepared according to the general procedure A from 2‐bromoanisol (5.0 g, 3.4 mL, 26.8 mmol, 3 equiv.), magnesium turnings (0.9 g, 35.6 mmol, 4 equiv.), BF_3_⋅Et_2_O (1.3 g, 8.9 mmol, 1 equiv.) and enantiopure α‐methylbenzylamine (10.8 g, 11.5 mL, 89.0 mmol, 10 equiv.). Crystallization of the product from ethanol afforded **2** **e** as a colorless, crystalline solid. Yield: 161.9 mg, 0.4 mmol, 4 %; ^1^H NMR (300 MHz, ACN‐*d_3_*): δ/ppm=7.38–7.33 (2H, m, C*H*‐ar), 7.29–7.27 (1H, m, C*H*‐ar), 7.23–7.21 (2H, m, C*H*‐ar), 7.10–7.04 (3H, m, C*H*‐ar), 7.01–6.98 (3H, m, C*H*‐ar), 6.78–6.70 (6H, m, C*H*‐ar), 6.63 (1H, m, N*H*), 6.39 (1H, s_br_, N*H*), 4.08–4.02 (1H, m, C*H*), 3.49 (9H, s, O‐C*H*_3_), 1.11 (3H, d, ^3^J_H−H_=6.8 Hz, C*H*_3_). ^13^C NMR (75 MHz, ACN‐*d_3_*): δ/ppm=163.44, 137.20, 129.75, 128.27, 127.35, 126.90, 120.78, 110.79, 55.08, 54.90, 22.89. ^11^B NMR (128 MHz, ACN‐*d_3_*): δ/ppm=−1.98.

**Synthesis of tris‐(*o‐*methoxyphenyl)‐borane 4‐methoxy‐α‐methylbenzyl amine complex (2** **f)**: Following the procedure reported for **2** **a**, compound **2** **f** was prepared according to the general procedure A from 2‐bromoanisol (10.0 g, 6.7 mL, 53.5 mmol, 3 equiv.), magnesium turnings (1.73 g, 71.1 mmol, 4 equiv.), BF_3_⋅Et_2_O (2.5 g, 2.2 mL, 17.7 mmol, 1 equiv.) and enantiopure 1‐(4‐methoxyphenyl)ethylamine (2.7 g, 2.6 mL, 17.7 mmol, 1 equiv.). Crystallization of the product from ethanol gave **2** **f** as a colorless, crystalline solid. Yield: 620.0 mg, 1.3 mmol, 7 %. ^1^H NMR (300 MHz, ACN‐*d_3_*): δ/ppm=7.15–7.04 (5H, m, C*H*‐ar), 7.00–6.97 (3H, m, C*H*‐ar), 6.89–6.85 (2H, m, C*H*‐ar), 6.78–6.69 (6H, m, C*H*‐ar), 6.44 (2H, s_br_, N*H*), 4.07‐3.97 (1H, s, C*H*), 3.77 (3H, s, O‐C*H*_3_), 3.51 (9H, s, O‐C*H*_3_), 1.12 (3H, d, ^3^J_H−H_=6.8 Hz, C*H*_3_). ^13^C NMR (75 MHz, ACN‐*d_3_*): δ/ppm=163.43, 159.91, 138.04, 137.18, 128.21, 127.33, 120.77, 115.00, 110.81, 55.97, 55.13, 54.22, 22.70. ^11^B NMR (128 MHz, ACN‐*d_3_*): δ/ppm=−2.09.

**NMR spectroscopy**: ^1^H‐ and ^13^C NMR spectra were recorded on a Bruker Avance‐III 300 MHz spectrometer, ^11^B{^1^H} spectra on a Bruker Avance‐III 400 MHz spectrometer. Standard abbreviations of signal multiplicities were used (s=singlet, s_br_=broad singlet, d=dublet, t=triplet, q=quartet, m=multiplet).

**Computational details**: Conformational searches for all investigated compounds were carried out systematically by preparing individual starting structures for unique combinations of torsional angles (threefold‐rotation). All calculations were carried out using Gaussian 09 Rev. E.01^[13]^ employing the B3LYP/6‐31+G(2d,p)/IEFPCM(CHCl_3_) level of DFT. IR and VCD spectra were simulated by assigning a uniform Lorentzian band shape of 6 cm^−1^ half‐width at half‐height to the computed dipole and rotational strengths. The vibrational spectra presented in the main text are scaled with a frequency scaling factor σ of 0.98. Relative zero‐point energy corrected energies, ΔE_ZPC_, were used to determine Boltzmann weights. 3D‐representations were made with Cylview.^[14]^ Note that we did not repeat the calculations on other levels of density functional theory, such as other functional families, or by incorporation of dispersion interactions as these types of calculations have led to worse results in our previous study on **1**. Similarity fitting of computed spectra to the experimental data was done by finding the coefficients *χ_I_* of the linear combination Σ(*χ*_*I ⋅*_ *VCD_i_*) of up to six spectra *VCD_i_* that gives maximum overlap. The populations of each considered conformer were varied in steps of 5 %.

**IR and VCD spectroscopy**: The IR and VCD spectra were recorded on a Bruker Vertex FTIR spectrometer equipped with a PMA 50 module for VCD measurements. The sample was held in a transmission cell with BaF_2_ windows and 100 μm path length. Concentration are given in the main text. Spectra were recorded at room temperature with 4 cm^−1^ spectral resolution by accumulating 32 scans for the IR and ∼16000 scans (4 h accumulation time) for VCD. Baseline correction of the VCD spectra was done by subtraction of the spectra of the solvent or the racemic mixture recorded under identical conditions.

Deposition Numbers 2205404 (2a), 2205402 (2e) and 2205403 (2 f) contain the supplementary crystallographic data for this paper. These data are provided free of charge by the joint Cambridge Crystallographic Data Centre and Fachinformationszentrum Karlsruhe Access Structures service.

## Conflict of interest

The authors declare no conflict of interest.

1

## Supporting information

As a service to our authors and readers, this journal provides supporting information supplied by the authors. Such materials are peer reviewed and may be re‐organized for online delivery, but are not copy‐edited or typeset. Technical support issues arising from supporting information (other than missing files) should be addressed to the authors.

Supporting Information

## Data Availability

The data that support the findings of this study are available from the corresponding author upon reasonable request.
