# Supplementary material for: Inducing Propeller Chirality in Triaryl Boranes with Chiral Amines
Source: Chemistry. 2022 Oct 25;28(70):e202202812. doi: 10.1002/chem.202202812 (PMC10092159; doi:10.1002/chem.202202812)
Supplement: Supplementary file 1 — Supporting Information [file CHEM-28-0-s001.pdf]

Chemistry–A European Journal

Supporting Information

Inducing Propeller Chirality in Triaryl Boranes with Chiral Amines

Michael Kemper, Sven Reese, Elric Engelage, and Christian Merten*

Table of Contents

1. CD spectroscopy 2
2. Additional figures 3
3. Conformational analysis 6
   Conformational analysis of 2a 6
   Conformational analysis of 2b 7
   Conformational analysis of 2c 7
   Conformational analysis of 2d 8
   Conformational analysis of 2e 9
   Conformational analysis of 2f 10
4. Crystallography 12
5. NMR spectra 27
6. Selected Cartesian coordinates 36
7. References 39

1. CD spectroscopy

UV-Vis and CD Spectra were recorded on an Applied Physics Chirascan^{TM}-plus CD Spectrometer at room temperature. The quartz cuvettes used had a path length of 1 cm. Concentrations are given in the caption of Figure S1. Spectra were acquired in the spectral range of 190 to 300 nm with a step size of 0.5 nm between measurement points and a sampling time of one second per point. Measurements were repeated eight times and averaged to reduce the signal-to-noise ratio.

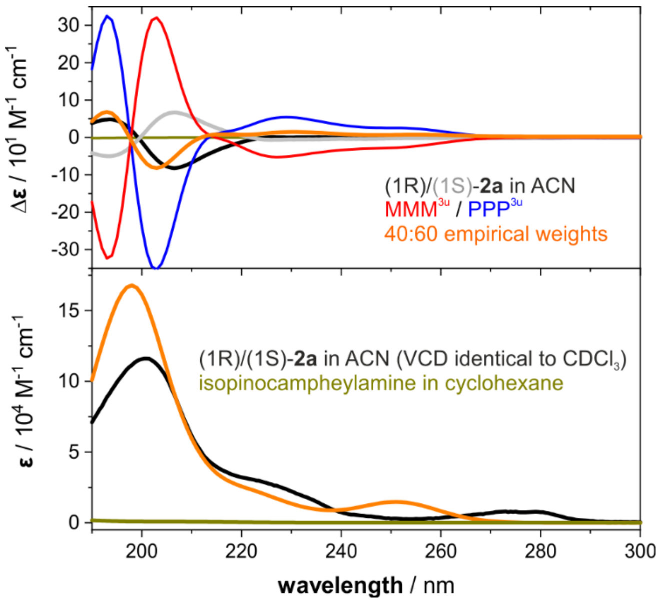

Figure S1. Experimental and computed UV and ECD spectra of **2a** (3.7 µM, 1 cm path length, ACN) and isopinocampheyl amine (0.27 mM, 1cm path length, cyclohexane) together with the calculated ECD spectra of *MMM*^{3u}- and *PPP*^{3u}-(1*R*)-**2a** and a combination of these two with empirical weights of 40:60. Simulations were performed on the TD-DFT B3LYP/6-31+G(2d,p)/IEFPCM(ACN) level of theory assuming 0.3 eV HWHH.

2. Additional figures

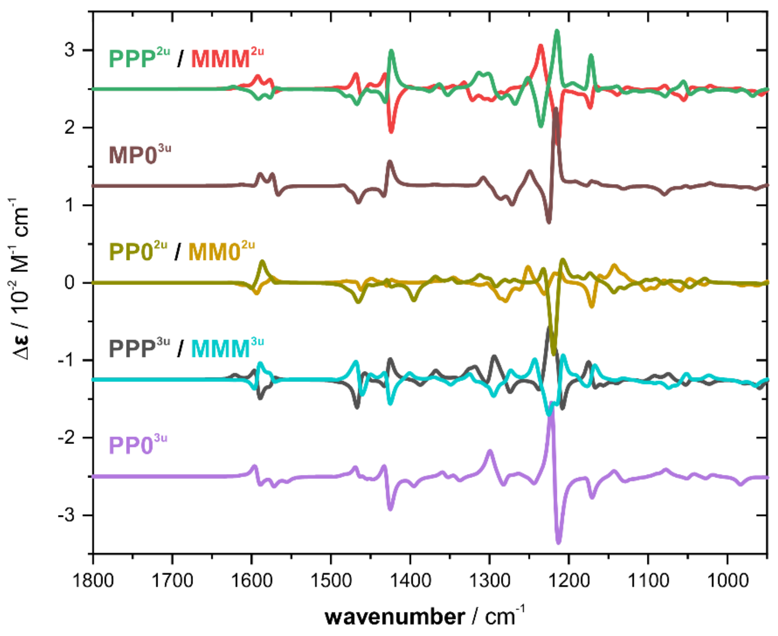

Figure S2. Single conformer spectra of **2b** (B3LYP/6-31+g(2d,p)/IEFPCM($CDCl_3$)).

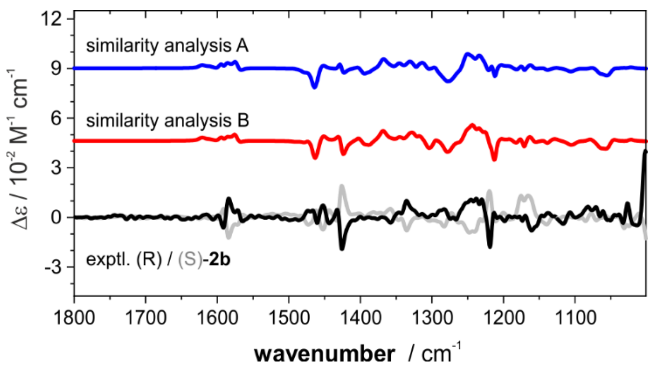

Figure S3. Two representative good fits for **2b** obtained from similarity fitting analysis. Composition similarity analysis A: 0.2 PPP^{3u} + 0.0 PP0^{3u} + 0.1 PPP^{2u} + 0.15 PP0^{2u} + 0.05 MMM^{3u} + 0.1 MM0^{3u} + 0.25 MMM^{2u} + 0.15 MM0^{2u}, M/P = 55:45. Composition similarity analysis B: 0.3 PPP^{3u} + 0.0 PP0^{3u} + 0.0 PPP^{2u} + 0.1 PP0^{2u} + 0.2 MMM^{3u} + 0.05 MM0^{3u} + 0.2 MMM^{2u} + 0.15 MM0^{2u}, M/P = 60:40.

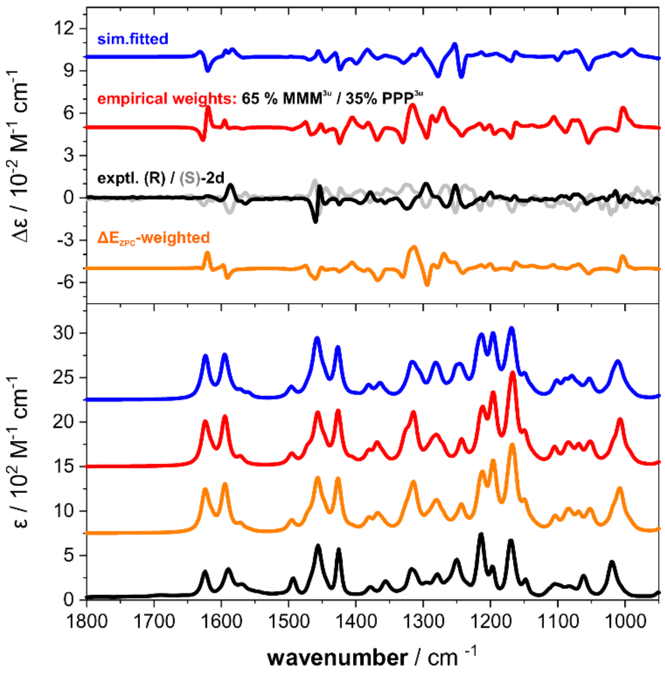

Figure S4. IR and VCD spectra of **2d**. Composition of the similarity-fitted spectrum: 0.15 PPP^{1u} + 0.25 PPP^{2u} + 0.5 MMM^{2u} + 0.1 MMM^{3u}

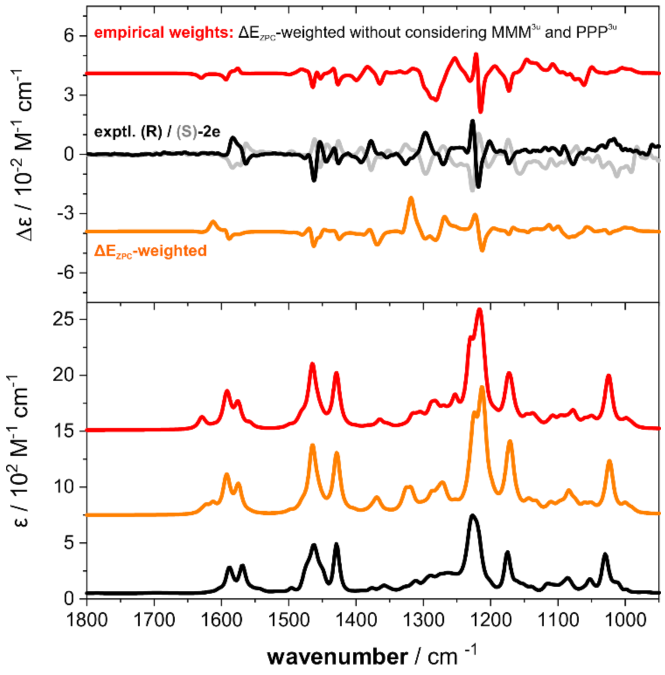

Figure S5. IR and VCD spectra of **2e**. Varying the ratio of MMM^{3u} and PPP^{3u} does not lead to any improvement, so as a test, the empirical weights were obtained by leaving MMM^{3u} and PPP^{3u} out of the conformational distribution.

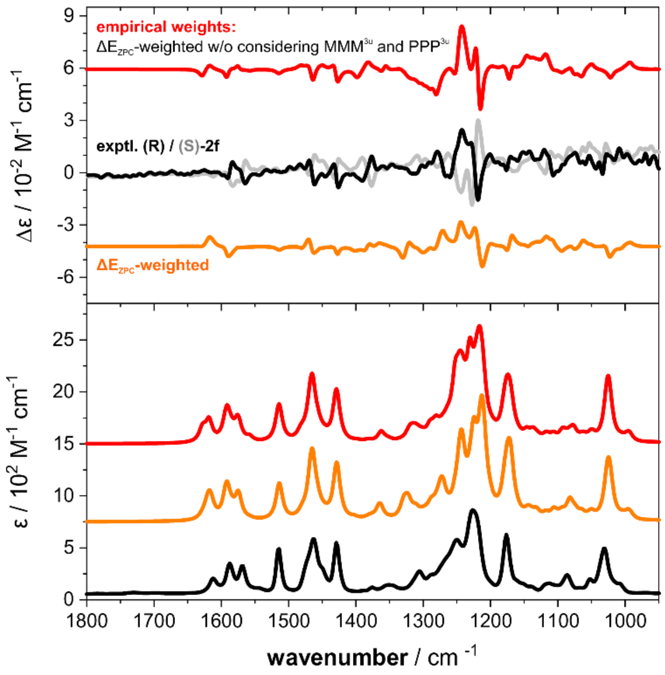

Figure S6. IR and VCD spectra of **2f**. Varying the ratio of MMM^{3u} and PPP^{3u} does not lead to any improvement, so as a test, the empirical weights were obtained by leaving MMM^{3u} and PPP^{3u} out of the conformational distribution.

3. Conformational analysis

Conformational analysis of 2a

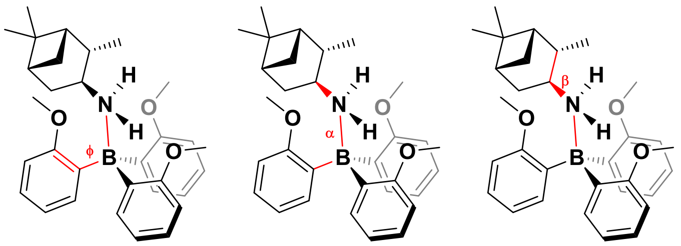

Table S1. Conformational analysis of **2a**.

	ϕ–1 [°]	ϕ–2 [°]	ϕ–3 [°]	α [°]	β [°]	ΔE_{ZPC} [kcal mol^{-1}]	ΔG_{298K} [kcal mol^{-1}]	$\chi(\Delta E_{ZPC})$ [%]	$\chi(\Delta G_{298K})$ [%]
**2a**-c1-MMM^{3u}	73.9	45.8	42.7	-50.834	153.1	0.0$^{a)}$	0.11	48.7	43.0
**2a**-c2-PPP^{3u}	-73.5	-43.8	-43.4	46.841	151.7	0.02	0.0$^{a)}$	47.4	51.4
**2a**-c3-MM0^{2u}	59.3	50.0	-179.3	62.397	158.8	1.75	1.82	2.5	2.4
**2a**-c4-MMM^{2u}	-109.8	46.1	40.4	-44.303	157.5	2.57	2.30	0.6	1.1
**2a**-c5-MP0^{3u}	77.3	3.9	-42.2	48.144	140.5	2.66	1.94	0.5	1.9
**2a**-c6-PP0^{3u}	-58.1	8.9	-48.5	-61.853	83.7	4.03	4.02	0.1	0.1
**2a**-c7-MP0^{1u}	77.5	-167.3	-38.9	45.759	140.1	3.91	3.56	0.1	0.1
**2a**-c8-PPP^{2u}	110.1	-42.1	-45.6	43.874	95.6	5.34	4.90	0.0	0.0
**2a**-c9-MP0^{1u}	-91.7	38.0	168.3	50.47	145.3	6.09	5.10	0.0	0.0
**2a**-c10-PP0^{1u}	176.8	128.7	-57.0	60.462	104.4	8.32	8.43	0.0	0.0
**2a**-c11-MMM^{1u}	-106.6	-157.6	40.7	-38.113	152.1	8.50	7.92	0.0	0.0
**2a**-c12-MMM^{3u}	83.9	40.1	40.7	-39.028	-58.7	9.18	9.29	0.0	0.0
**2a**-c13-PP0^{2u}	-177.4	-49.0	-62.0	75.259	-59.0	9.89	9.85	0.0	0.0
**2a**-c14-MM0^{1u}	-112.7	47.8	-178.5	62.311	-121.6	9.89	10.04	0.0	0.0
**2a**-c15-PP0^{0u}	177.3	133.5	109.6	66.713	108.1	14.49	13.86	0.0	0.0
**2a**-c16-PP0^{0u}	122.3	107.0	-179.4	29.483	161.2	14.68	13.64	0.0	0.0
**2a**-c17-PP0^{1u}	-179.5	132.9	-61.3	76.67	-58.0	15.99	15.72	0.0	0.0
**2a**-c18-MM0^{0u}	-115.2	-134.7	-175.4	58.51	-119.1	16.86	16.77	0.0	0.0

$^{a)}$ referenced to E_{ZPC}(**2a**-c1-MMM^{3u}) = -1510.120274 hartree and G_{298K}(**2a**-c2-PPP^{3u}) = -1510.185778 hartree.

Theoretically, both lone pairs of the methoxy oxygens could act as hydrogen bond acceptor. However, none of the starting structures in which we rotated the OMe group out of plane to allow for an interaction with the respective other lone pair lead to a stable conformer. Instead, the methoxy group either rotated back into the plane of the phenyl ring or the blade with the rotated methoxy group flipped from M to P and *vice versa*.

Conformational analysis of 2b

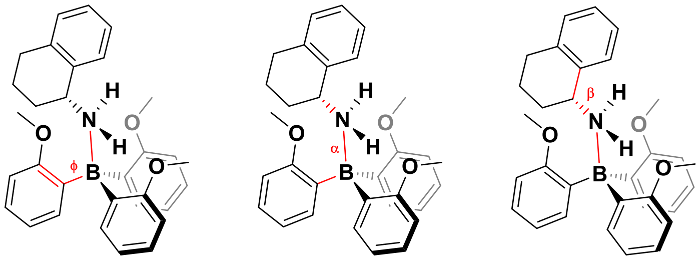

Table S2. Conformational analysis of **2b**.

	ϕ–1 [°]	ϕ–2 [°]	ϕ–3 [°]	α [°]	β [°]	ΔE_{ZPC} [kcal mol^{-1}]	ΔG_{298K} [kcal mol^{-1}]	$\chi(\Delta E_{ZPC})$ [%]	$\chi(\Delta G_{298K})$ [%]
**2b**-c1-PP0^{3u}	15.8	-40.5	-60.1	92.2	172.5	0.0[a)]	0.0[a)]	69.5	66.4
**2b**-c2-PPP^{3u}	-71.1	-46.1	-44.3	55.9	147.1	0.91	0.75	15.0	18.6
**2b**-c3-MMM^{3u}	44.3	44.1	73.0	79.8	154.2	0.95	0.92	14.0	14.0
**2b**-c4-MM0^{2u}	60.8	48.6	178.9	57.0	137.2	2.47	2.85	1.1	0.5
**2b**-c5-PP0^{2u}	179.7	-49.3	-58.6	88.8	168.4	3.36	3.66	0.2	0.1
**2b**-c6-MM0^{3u}	70.8	11.5	-39.1	56.4	132.2	3.49	3.59	0.2	0.2
**2b**-c7-MMM^{2u}	45.5	40.8	-110.6	81.4	159.0	4.10	3.86	0.1	0.1
**2b**-c8-PP0^{2u}	100.0	-31.8	-44.3	49.0	139.5	4.65	4.70	0.0	0.0

[a)] referenced to E_{ZPC}(**2b**-c1-PP0^{3u}) = -1506.608247 hartree and G_{298K}(**2b**-c1-PP0^{3u}) = -1506.673909 hartree.

Conformational analysis of 2c

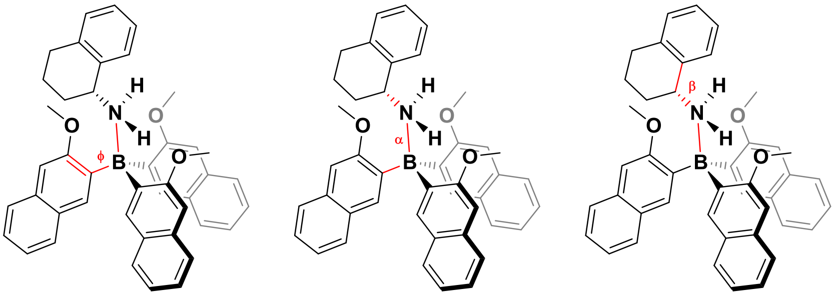

Table S3. Conformational analysis of **2c**.

	ϕ–1 [°]	ϕ–2 [°]	ϕ–3 [°]	α [°]	β [°]	ΔE_{ZPC} [kcal mol^{-1}]	ΔG_{298K} [kcal mol^{-1}]	$\chi(\Delta E_{ZPC})$ [%]	$\chi(\Delta G_{298K})$ [%]
**2c**-c1-PP0^{3u}	15.8	–41.7	–59.8	91.7	172.4	0.00	0.00	63.3	59.9
**2c**-c2-PPP^{3u}	-44.4	-70.3	-47.2	-69.7	147.6	0.66	0.47	20.6	27.2
**2c**-c3-MMM^{3u}	44.4	44.0	72.5	79.6	154.1	0.89	0.97	14.0	11.6
**2c**-c4-MM0^{2u}	61.5	48.8	178.7	55.5	136.6	2.37	2.91	1.2	0.4
**2c**-c5-MM0^{3u}	71.2	9.0	-41.1	57.0	130.4	3.24	3.09	0.3	0.3
**2c**-c6-PP0^{2u}	179.5	-49.5	-59.0	89.5	168.4	3.24	3.22	0.3	0.3
**2c**-c7-MM0^{2u}	46.4	43.1	-112.4	80.0	160.1	3.29	3.91	0.2	0.1
**2c**-c8-MP0^{2u}	-165.0	-37.9	72.8	175.2	132.0	3.78	3.81	0.1	0.1
**2c**-c9-PPP^{2u}	-36.0	-46.7	104.5	169.5	146.6	4.10	4.21	0.1	0.0
**2c**-c10-PP0^{1u}	130.4	-58.6	175.0	-154.3	168.1	4.32	4.15	0.0	0.1
**2c**-c11-MM0^{0u}	170.4	140.8	-75.1	-172.1	138.2	7.81	8.20	0.0	0.0
**2c**-c12-MM0^{1u}	-175.2	76.7	-139.5	-171.6	158.7	10.15	10.64	0.0	0.0
**2c**-c13-PP0^{0u}	116.5	112.3	176.3	32.2	153.9	10.73	11.10	0.0	0.0

[a)] referenced to E_{ZPC}(**2c**-c1-PP0^{3u}) = -1967.434833 hartree and G_{298K}(**2c**-c1-PP0^{3u}) = -1967.510310 Hartree.

Conformational analysis of 2d

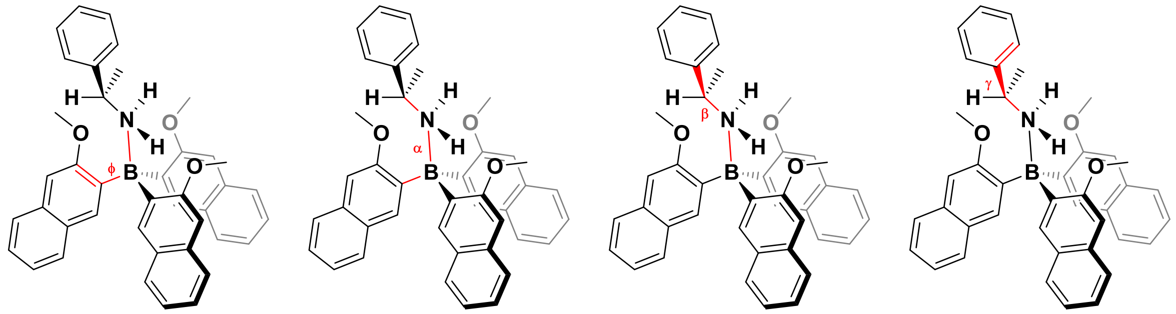

Table S4. Conformational analysis of **2d**.

	ϕ–1 [°]	ϕ–2 [°]	ϕ–3 [°]	α [°]	β [°]	γ [°]	ΔE_{ZPC} [kcal mol^{-1}]	ΔG_{298K} [kcal mol^{-1}]	$\chi(\Delta E_{ZPC})$ [%]	$\chi(\Delta G_{298K})$ [%]
**2d**-c1-MMM^{3u}	45.3	71.8	43.3	-168.2	156.0	64.2	0.00$^{a)}$	0.02	39.9	41.1
**2d**-c2-PPP^{3u}	-47.3	-42.5	-70.6	177.8	142.9	55.7	0.01	0.00$^{a)}$	39.3	42.5
**2d**-c3-MM0^{2u}	59.5	49.8	-179.0	58.3	135.1	48.3	0.66	0.95	13.1	8.6
**2d**-c4-PP0^{3u}	13.5	-44.2	-59.8	91.3	169.3	66.8	1.49	1.57	3.2	3.0
**2d**-c5-MM0^{3u}	60.9	46.1	-12.8	57.6	147.6	40.3	2.00	2.23	1.4	1.0
**2d**-c6-PP0^{2u}	179.4	-49.6	-58.1	88.2	168.9	-111.4	2.17	2.28	1.0	0.9
**2d**-c7-MMM^{3u}	43.3	44.0	71.2	66.4	86.9	19.9	2.34	2.55	0.8	0.6
**2d**-c8-MP0^{3u}	68.5	16.5	-35.3	60.5	136.7	53.8	2.58	2.22	0.5	1.0
**2d**-c9-MMM^{2u}	44.7	46.7	-115.4	74.9	160.8	61.4	2.94	2.79	0.3	0.4
**2d**-c10-MP0^{2u}	-165.4	-37.2	73.2	177.3	133.1	58.8	3.23	2.65	0.2	0.5
**2d**-c11-PPP^{2u}	-42.2	-45.1	109.7	178.5	138.6	57.0	3.57	3.39	0.1	0.1
**2d**-c12-PP0^{2u}	-48.3	167.0	-78.6	176.7	143.2	54.3	3.37	3.02	0.1	0.3
**2d**-c13-MM0^{2u}	177.0	81.6	44.2	-170.2	157.8	65.6	3.99	4.22	0.0	0.0
**2d**-c14-MM0^{1u}	-128.9	-175.8	58.6	179.8	132.7	47.6	5.62	5.79	0.0	0.0
**2d**-c15-PP0^{1u}	129.9	-57.7	175.0	-155.5	166.0	63.3	6.67	6.67	0.0	0.0
**2d**-c16-PPP^{3u}	-68.6	-47.2	-46.0	60.5	-77.8	-13.5	8.68	9.58	0.0	0.0
**2d**-c17-MP0^{3u}	36.6	-13.0	-65.3	73.1	-59.3	83.8	9.30	9.86	0.0	0.0
**2d**-c18-MM0^{1u}	-175.1	76.7	-139.9	-172.8	157.2	68.3	10.08	10.41	0.0	0.0
**2d**-c19-MM0^{0u}	-142.8	172.9	-96.9	176.5	132.5	53.9	11.34	11.17	0.0	0.0
**2d**-c20-PP0^{0u}	179.6	138.7	104.9	93.3	165.0	-112.5	12.10	12.24	0.0	0.0
**2d**-c21-PP0^{0u}	117.0	113.1	175.1	41.4	144.6	53.1	14.38	14.22	0.0	0.0

$^{a)}$ referenced to E_{ZPC}(**2d**-c1-MMM^{3u}) = -1890.045102 hartree and G_{298K}(**2d**-c2-PPP^{3u}) = -1890.119466 hartree.

Conformational analysis of 2e

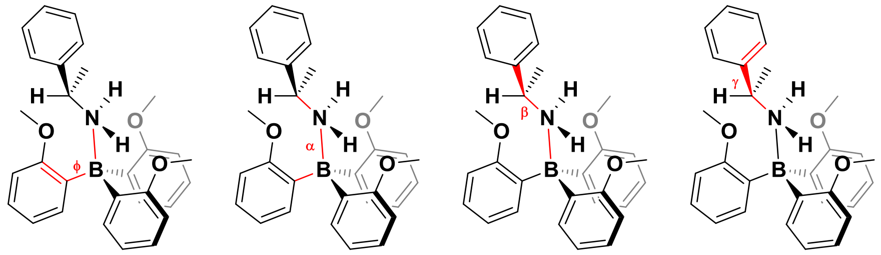

Table S5. Conformational analysis of **2e**.

	ϕ–1 [°]	ϕ–2 [°]	ϕ–3 [°]	α [°]	β [°]	γ [°]	ΔE_{ZPC} [kcal mol^{-1}]	ΔG_{298K} [kcal mol^{-1}]	$\chi(\Delta E_{ZPC})$ [%]	$\chi(\Delta G_{298K})$ [%]
**2e**-c1-MMM^{3u}	43.2	44.6	72.7	77.1	155.5	61.5	0.00	0.08	40.5	39.8
**2e**-c2-PPP^{3u}	-71.6	-46.1	-42.6	58.7	142.8	55.6	0.02	0.00	39.4	45.8
**2e**-c3-MM0^{2u}	59.2	49.3	-179.8	59.5	135.9	49.6	0.79	1.23	10.8	5.8
**2e**-c4-PP0^{3u}	13.1	-43.8	-59.9	92.0	169.5	66.3	1.33	1.42	4.3	4.2
**2e**-c5-MM0^{3u}	61.1	45.1	-12.8	58.1	147.9	40.0	1.87	1.93	1.7	1.8
**2e**-c6-PP0^{2u}	178.8	-50.1	-58.7	63.0	97.9	-124.1	2.05	2.84	1.3	0.4
**2e**-c7-PP0^{2u}	179.5	-49.6	-57.9	87.8	168.0	-110.9	2.34	2.60	0.8	0.6
**2e**-c8-MMM^{3u}	43.2	43.4	72.0	66.7	86.8	20.1	2.46	3.07	0.6	0.3
**2e**-c9-MMM^{2u}	43.8	44.6	-114.1	76.5	159.7	62.8	3.18	2.76	0.2	0.4
**2e**-c10-PP0^{3u}	8.2	-48.4	-59.6	65.7	85.2	63.7	3.23	4.15	0.2	0.0
**2e**-c11-PPP^{2u}	96.3	-27.1	-43.0	48.1	139.0	57.2	3.59	2.68	0.1	0.5
**2e**-c12-PPP^{2u}	108.6	-41.2	-43.2	51.8	140.3	-125.5	3.62	3.22	0.1	0.2
**2e**-c13-MP0^{3u}	66.3	12.3	-38.3	45.3	82.1	71.3	4.06	3.64	0.0	0.1
**2e**-c14-MM0^{2u}	81.0	43.8	176.9	-51.6	156.5	65.6	4.10	3.61	0.0	0.1
**2e**-c15-PPP^{2u}	107.0	-37.6	-46.5	38.6	86.5	57.6	5.55	5.70	0.0	0.0
**2e**-c16-MMM^{2u}	46.6	49.0	-122.1	55.8	96.2	41.8	5.58	5.68	0.0	0.0
**2e**-c17-MM0^{1u}	57.8	-129.9	-176.8	57.0	132.9	49.6	6.36	6.32	0.0	0.0
**2e**-c18-PP0^{1u}	176.4	130.5	-57.3	90.8	167.0	65.4	7.30	7.31	0.0	0.0
**2e**-c19-PP0^{1u}	175.5	128.3	-57.0	63.3	100.3	-125.3	7.70	8.50	0.0	0.0
**2e**-c20-PPP^{3u}	-72.4	-43.8	-45.0	56.6	-71.8	-28.5	8.89	9.72	0.0	0.0
**2e**-c21-PP0^{2u}	178.5	-50.0	-59.5	77.1	-56.8	-104.5	9.09	9.71	0.0	0.0
**2e**-c22-MM0^{3u}	41.1	44.0	80.5	77.8	-80.5	66.2	9.43	9.95	0.0	0.0
**2e**-c23-MP0^{3u}	33.6	-16.4	-64.3	74.0	-58.9	81.7	9.45	9.82	0.0	0.0
**2e**-c24-MM0^{1u}	-140.3	-176.2	76.5	73.4	157.9	-110.6	10.81	10.95	0.0	0.0
**2e**-c25-MM0^{0u}	-140.2	175.4	-98.4	177.2	133.6	-129.9	11.62	11.50	0.0	0.0
**2e**-c26-PP0^{0u}	-179.2	138.3	104.9	92.5	166.2	-105.9	12.37	12.51	0.0	0.0
**2e**-c27-PP0^{0u}	-60.6	-156.2	-168.6	25.7	0.0	53.4	14.72	14.41	0.0	0.0

[a] referenced to E_{ZPC}(**2e**-c1-MMM^{3u}) = -1429.218320 hartree and G_{298K}(**2e**-c2-PPP^{3u}) = -1429.282620 hartree.

Conformational analysis of 2f

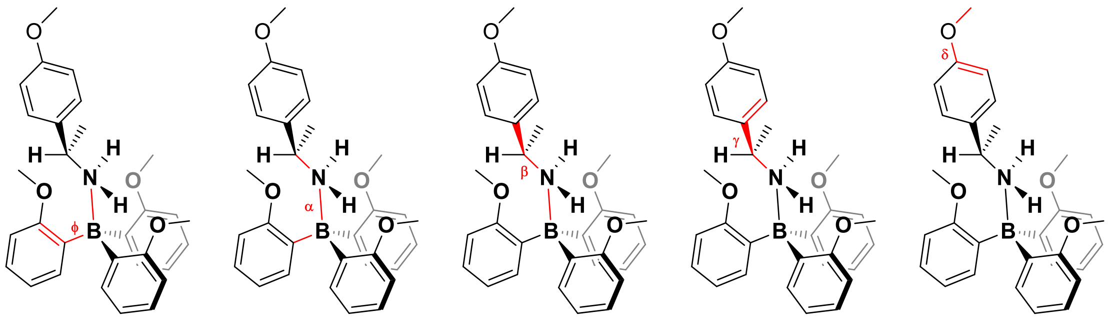

Table S6. Conformational analysis of **2f**.

	ϕ–1 [°]	ϕ–2 [°]	ϕ–3 [°]	α [°]	β [°]	γ [°]	δ [°]	ΔE_{ZPC} [kcal mol^{-1}]	ΔG_{298K} [kcal mol^{-1}]	$\chi(\Delta E_{ZPC})$ [%]	$\chi(\Delta G_{298K})$ [%]
**2f**-c1-PPP^{3u}	-71.5	-45.9	-43.1	57.6	143.2	59.4	179.9	0.00	0.00	21.6	24.6
**2f**-c2-MMM^{3u}	43.2	44.5	72.9	77.3	154.8	65.9	179.8	0.03	0.28	20.6	15.2
**2f**-c3-PPP^{3u}	-71.6	-46.4	-42.5	58.2	142.9	57.9	179.9	0.11	0.05	18.0	22.5
**2f**-c4-MMM^{3u}	43.2	45.1	72.5	77.0	155.4	67.3	0.2	0.12	0.13	17.7	19.6
**2f**-c5-MM0^{2u}	59.4	48.9	-179.6	59.0	135.6	-130.6	-0.3	0.68	0.94	6.8	5.0
**2f**-c6-MM0^{2u}	59.2	49.2	-179.2	58.7	134.7	-131.9	-179.9	0.80	0.93	5.5	5.1
**2f**-c7-PP0^{3u}	124.6	-113.2	-13.9	-110.5	-87.2	67.8	179.8	1.22	1.33	2.7	2.6
**2f**-c8-PP0^{3u}	124.7	-113.3	-13.6	-110.6	-87.0	68.3	0.1	1.38	1.61	2.1	1.6
**2f**-c9-PP0^{2u}	149.4	-110.1	-144.0	76.1	106.1	-122.4	-0.7	1.87	2.68	0.9	0.3
**2f**-c10-MM0^{3u}	60.8	44.8	-13.8	59.2	151.2	-137.7	-0.7	2.02	2.17	0.7	0.6
**2f**-c11-MM0^{3u}	60.8	46.0	-12.5	58.5	149.2	-143.3	-179.3	2.04	1.94	0.7	0.9
**2f**-c12-PP0^{2u}	149.1	-110.0	-144.2	76.4	105.9	-122.8	180.0	2.19	3.14	0.5	0.1
**2f**-c13-PP0^{2u}	179.3	-49.3	-57.8	88.1	169.2	-109.2	0.4	2.28	2.87	0.5	0.2
**2f**-c14-PP0^{2u}	179.5	-49.5	-57.8	87.9	169.3	-107.7	179.8	2.33	2.51	0.4	0.4
**2f**-c15-MMM^{3u}	109.5	-88.9	87.8	-97.1	-178.6	19.7	-1.5	2.63	3.36	0.3	0.1
**2f**-c16-MMM^{3u}	109.6	-88.9	88.0	-97.1	-178.5	21.8	-179.8	2.83	3.39	0.2	0.1
**2f**-c17-PP0^{3u}	128.6	-113.7	-6.9	-113.3	169.7	64.8	179.6	3.10	4.34	0.1	0.0
**2f**-c18-MMM^{2u}	109.4	-87.9	-108.9	-98.9	-100.5	65.4	179.6	3.16	3.15	0.1	0.1
**2f**-c19-MMM^{2u}	109.4	-87.8	-109.7	-99.0	-100.2	65.4	0.4	3.28	3.17	0.1	0.1
**2f**-c20-PP0^{3u}	128.0	-113.5	-8.1	-112.8	169.3	65.0	0.1	3.42	4.86	0.1	0.0
**2f**-c21-PPP^{2u}	96.1	-92.9	-66.6	-56.9	-166.1	59.5	179.7	3.50	2.62	0.1	0.3
**2f**-c22-PPP^{2u}	97.1	-92.4	-78.9	-45.8	-171.4	-122.4	-0.8	3.66	3.56	0.0	0.1
**2f**-c23-PPP^{2u}	96.2	-93.0	-66.2	-57.4	-165.4	59.4	-0.2	3.70	2.75	0.0	0.2
**2f**-c24-PPP^{2u}	105.4	-38.1	-42.9	48.7	140.2	-121.3	-179.9	3.79	3.22	0.0	0.1
**2f**-c25-PP0^{2u}	168.0	-77.9	-47.6	-67.7	143.3	-124.2	0.0	3.81	3.83	0.0	0.0
**2f**-c26-PP0^{2u}	168.3	-78.1	-47.7	-67.6	143.1	-125.7	179.7	3.86	3.80	0.0	0.0
**2f**-c27-MM0^{2u}	81.5	43.8	176.2	-51.9	156.0	-110.7	0.0	3.95	3.93	0.0	0.0
**2f**-c28-MM0^{2u}	81.5	43.9	176.2	-52.0	156.2	-109.3	-179.9	4.15	4.25	0.0	0.0
**2f**-c29-PP0^{2u}	178.4	-44.8	-79.3	179.8	137.8	-119.9	-0.3	4.28	4.30	0.0	0.0
**2f**-c30-PP0^{2u}	178.4	-44.7	-79.4	180.0	138.0	-118.8	-179.8	4.44	4.49	0.0	0.0
**2f**-c31-MM0^{2u}	-160.9	44.5	78.4	81.4	149.3	-111.7	-0.3	5.07	5.38	0.0	0.0

**2f**-c32-MM0[2u]	44.6	78.1	-159.9	-163.8	150.4	-110.4	-179.6	5.22	5.60	0.0	0.0
**2f**-c33- MM0[2u]	-161.0	44.6	78.3	81.5	149.8	-108.9	-179.5	5.26	5.80	0.0	0.0
**2f**-c34-MM0[1u]	58.3	-130.0	-177.5	57.4	133.2	-131.9	-180.0	6.42	7.06	0.0	0.0
**2f**-c35-MM0[1u]	58.3	-129.8	-177.3	57.1	132.8	-132.1	-0.1	6.44	7.27	0.0	0.0
**2f**-c36-PP0[1u]	-57.6	177.1	129.0	-60.6	99.1	-123.3	-0.1	7.54	8.68	0.0	0.0
**2f**-c37-PP0[1u]	177.0	129.1	-57.6	62.9	99.1	-123.5	-179.5	7.88	8.97	0.0	0.0
**2f**-c38-MM0[1u]	49.6	-112.9	-169.7	179.9	97.1	-130.3	0.5	9.46	9.83	0.0	0.0
**2f**-c39-MM0[1u]	49.9	-113.5	-169.7	179.4	97.0	-132.0	179.8	9.66	10.15	0.0	0.0
**2f**-c40-PP0[1u]	-74.8	170.1	141.5	73.2	132.3	-122.6	-0.2	10.31	10.35	0.0	0.0
**2f**-c41-PP0[1u]	-74.8	171.1	141.6	73.0	132.3	-121.7	179.6	10.38	10.72	0.0	0.0
**2f**-c42-MM0[1u]	-140.6	-177.1	77.0	73.8	157.4	-108.4	-180.0	10.63	10.17	0.0	0.0
**2f**-c43-MM0[1u]	-140.8	-177.1	77.1	74.2	158.1	-105.3	0.3	10.64	10.85	0.0	0.0
**2f**-c44-MM0[0u]	-96.4	-143.4	172.6	56.3	133.3	-125.5	-0.1	11.57	11.68	0.0	0.0
**2f**-c45-MM0[0u]	-96.4	-143.3	172.6	56.9	133.8	-126.4	-180.0	11.62	11.52	0.0	0.0
**2f**-c46-PP0[0u]	117.1	112.4	175.9	41.1	146.1	-123.3	-0.2	14.53	13.91	0.0	0.0
**2f**-c47-PP0[0u]	113.7	116.7	171.8	44.6	142.8	-125.2	-179.8	14.76	14.58	0.0	0.0
**2f**-c48-MM0[0u]	-110.9	-120.3	-175.7	-170.5	95.5	-129.4	0.5	16.57	16.54	0.0	0.0

[a)] referenced to E_{ZPC}(**2f**-c1-PPP[3u]) = -1543.720598 hartree and G_{298K}(**2f**-c1-PPP[3u]) = -1543.788609 hartree.

4. Crystallography

The crystal structure was analysed on a Rigaku Synergy dual source device, with Cu micro focus sealed tube (Cu Kα) using mirror monochromators and a HyPix-6000HE: Hybrid photon counting X-ray detector. The crystal was mounted in a Hampton CryoLoops using GE/Bayer silicone grease. Data was recorded and reduced using the CrysalisPro Software.[3] The structure was solved using WinGX[4] in combination with ShelXT[5] and refined with shelXle[6] and ShelXL.

Table S7. Crystal data and structure refinement for **2a**.

CCDC-Nr	2205404
Empirical formula	C31 H40 B N O3
Formula weight [g/mol]	485.45
Crystal system	Monoclinic
Space group	$P2_1$ (4)
Lattice parameters [Å]	
a	16.37279(10)
b	11.77682(4)
c	16.54338(11)
α	90
β	118.4847(8)
γ	90
Density [g/cm3]	1.15
Crystal size [mm3]	0.161 x 0.128 x 0.117
Volume [Å3]	2803.73(3)
Z	4
Temperature [K]	170.00(10)
Diffraction Device	XtaLAB Synergy, Dualflex, HyPix
Radiation Type	1.54184 Å (Cu K/ micro-focus sealed X-ray tube)
F(000)	1048
Absorption coefficient [mm-1]	0.562
Absorption correction	Gaussian
Measurement range	3.0 - 66.5
Index range	-19 < h < 19
	-14 < k < 14
	-19 < l < 19
Measured reflexes	128553
Independent	9882
Observed	9606
R(int)	0.0383
Completeness (%) / theta (°)	99.9 / 66.494
Transmission (min / max)	0.672 / 1.000
R1 (observed/all)	0.0357 / 0.0366
wR2 (observed/all)	0.0985 / 0.0996
GooF = S	1.049
Rest electron density max./min. [e-/Å³]	-0.241 / 0.476

Table S8. Atomic coordinates and equivalent isotropic displacement parameters [Å^2] for **2a**

	x	y	z	U(eq)	S.O.F.
O(1)	0.63567(12)	0.14529(19)	0.45863(13)	0.0537(5)	1
O(2)	0.95078(10)	0.19728(14)	0.54778(12)	0.0411(4)	1
O(3)	0.69090(13)	0.45107(14)	0.29785(12)	0.0457(4)	1
N(1)	0.77841(13)	0.29277(16)	0.49010(13)	0.0341(4)	1
C(1)	0.64931(14)	0.20887(18)	0.33040(15)	0.0331(4)	1
C(2)	0.60528(16)	0.2221(2)	0.23511(16)	0.0404(5)	1
C(3)	0.51227(19)	0.1965(3)	0.1777(2)	0.0545(7)	1
C(4)	0.45943(18)	0.1553(3)	0.2154(2)	0.0599(8)	1
C(5)	0.49910(18)	0.1387(3)	0.3086(2)	0.0556(7)	1
C(6)	0.59227(16)	0.1640(2)	0.36472(17)	0.0415(5)	1
C(7)	0.5858(3)	0.0892(4)	0.4970(3)	0.0852(12)	1
C(8)	0.81601(14)	0.10983(18)	0.43006(15)	0.0325(4)	1
C(9)	0.77352(16)	0.0081(2)	0.38618(17)	0.0392(5)	1
C(10)	0.8166(2)	-0.0972(2)	0.4123(2)	0.0505(6)	1
C(11)	0.9054(2)	-0.1043(2)	0.4860(2)	0.0515(6)	1
C(12)	0.95100(17)	-0.0064(2)	0.53214(17)	0.0428(5)	1
C(13)	0.90673(15)	0.09739(19)	0.50342(16)	0.0348(5)	1
C(14)	1.04042(16)	0.1889(2)	0.62623(17)	0.0451(5)	1
C(15)	0.80284(15)	0.30524(19)	0.33693(15)	0.0347(5)	1
C(16)	0.87536(16)	0.2606(2)	0.32439(16)	0.0396(5)	1
C(17)	0.90794(18)	0.3117(2)	0.26942(19)	0.0494(6)	1
C(18)	0.8689(2)	0.4110(3)	0.2243(2)	0.0552(7)	1
C(19)	0.7964(2)	0.4597(2)	0.23405(19)	0.0493(6)	1
C(20)	0.76426(17)	0.4077(2)	0.28893(17)	0.0400(5)	1
C(21)	0.6425(3)	0.5466(3)	0.2450(2)	0.0631(8)	1
C(22A)	0.75309(16)	0.4135(2)	0.49758(15)	0.0369(5)	0.608(3)
C(23A)	0.8310(4)	0.4985(5)	0.4956(4)	0.0418(10)	0.608(3)
C(24A)	0.8631(3)	0.5889(4)	0.5713(3)	0.0452(9)	0.608(3)
C(25A)	0.7788(4)	0.6437(4)	0.5758(4)	0.0450(11)	0.608(3)
C(26A)	0.7866(5)	0.5384(4)	0.6356(3)	0.0537(12)	0.608(3)
C(27A)	0.7365(4)	0.4321(5)	0.5785(5)	0.0455(14)	0.608(3)
C(28A)	0.6338(5)	0.4272(6)	0.5512(5)	0.0680(18)	0.608(3)
C(29A)	0.8883(4)	0.5315(4)	0.6625(3)	0.0657(12)	0.608(3)
C(30A)	0.8074(5)	0.7527(5)	0.6343(4)	0.0756(15)	0.608(3)
C(31A)	0.6951(13)	0.6707(19)	0.4887(16)	0.055(3)	0.608(3)
C(22B)	0.75309(16)	0.4135(2)	0.49758(15)	0.0369(5)	0.392(3)
C(23B)	0.8319(7)	0.4850(8)	0.5287(6)	0.0418(10)	0.392(3)
C(24B)	0.8424(5)	0.5705(6)	0.6043(5)	0.0452(9)	0.392(3)
C(25B)	0.7490(7)	0.6185(8)	0.5851(6)	0.0450(11)	0.392(3)
C(26B)	0.7350(7)	0.5101(7)	0.6274(5)	0.0537(12)	0.392(3)
C(27B)	0.7012(7)	0.4071(10)	0.5638(9)	0.0455(14)	0.392(3)
C(28B)	0.5985(9)	0.3993(10)	0.5086(9)	0.0680(18)	0.392(3)
C(29B)	0.8406(6)	0.5042(7)	0.6840(6)	0.0657(12)	0.392(3)
C(30B)	0.7584(9)	0.7267(8)	0.6441(7)	0.0756(15)	0.392(3)
C(31B)	0.675(2)	0.655(3)	0.485(3)	0.055(3)	0.392(3)

B(2)	0.76072(16)	0.2318(2)	0.39331(17)	0.0311(5)	1
O(4)	0.04354(10)	0.80374(13)	-0.04579(11)	0.0402(4)	1
O(5)	0.29773(12)	0.53700(14)	0.20040(12)	0.0438(4)	1
O(6)	0.35221(12)	0.83971(16)	0.03494(12)	0.0464(4)	1
N(2)	0.20832(13)	0.69470(16)	0.00983(12)	0.0321(4)	1
C(32)	0.18146(14)	0.88373(17)	0.07344(14)	0.0298(4)	1
C(33)	0.22620(15)	0.9826(2)	0.12035(16)	0.0355(5)	1
C(34)	0.18795(16)	1.0903(2)	0.09491(18)	0.0420(5)	1
C(35)	0.10195(17)	1.1031(2)	0.01848(18)	0.0424(5)	1
C(36)	0.05347(16)	1.0086(2)	-0.03029(17)	0.0388(5)	1
C(37)	0.09257(15)	0.90128(19)	-0.00162(16)	0.0333(4)	1
C(38)	-0.04379(16)	0.8166(2)	-0.12552(17)	0.0460(6)	1
C(39)	0.19092(15)	0.68839(18)	0.16498(14)	0.0316(4)	1
C(40)	0.12032(15)	0.7358(2)	0.17862(16)	0.0361(5)	1
C(41)	0.08753(18)	0.6877(2)	0.23468(17)	0.0444(6)	1
C(42)	0.12654(19)	0.5885(2)	0.28064(18)	0.0485(6)	1
C(43)	0.19578(19)	0.5368(2)	0.26926(16)	0.0445(6)	1
C(44)	0.22756(15)	0.58562(19)	0.21256(15)	0.0360(5)	1
C(45)	0.3421(3)	0.4383(3)	0.2510(2)	0.0625(8)	1
C(46)	0.34494(14)	0.77961(18)	0.16750(15)	0.0327(4)	1
C(47)	0.39090(16)	0.7681(2)	0.26275(16)	0.0404(5)	1
C(48)	0.48405(18)	0.7964(3)	0.31669(19)	0.0528(6)	1
C(49)	0.53440(18)	0.8365(3)	0.2766(2)	0.0587(7)	1
C(50)	0.49191(17)	0.8511(2)	0.1820(2)	0.0499(6)	1
C(51)	0.39859(15)	0.8238(2)	0.12940(17)	0.0381(5)	1
C(52)	0.4026(3)	0.8840(4)	-0.0072(3)	0.0836(12)	1
C(53)	0.22506(15)	0.57190(19)	-0.00220(15)	0.0345(5)	1
C(54)	0.32846(16)	0.5498(2)	0.02678(18)	0.0467(5)	1
C(55)	0.34144(18)	0.4796(2)	-0.0435(2)	0.0506(6)	1
C(56)	0.2844(3)	0.5348(3)	-0.1382(2)	0.0707(9)	1
C(57)	0.2017(2)	0.4651(2)	-0.14544(17)	0.0495(6)	1
C(58)	0.15503(17)	0.5345(2)	-0.10139(16)	0.0433(5)	1
C(59)	0.07003(18)	0.4761(3)	-0.1037(2)	0.0650(8)	1
C(60)	0.27422(17)	0.3772(2)	-0.0790(2)	0.0475(6)	1
C(61)	0.2564(2)	0.3095(2)	-0.0112(2)	0.0614(7)	1
C(62)	0.3007(3)	0.2932(3)	-0.1337(3)	0.0821(11)	1
B(1)	0.23300(16)	0.7586(2)	0.10741(16)	0.0301(5)	1
H(2)	0.640891	0.250132	0.207885	0.048	1
H(3)	0.485553	0.207385	0.113155	0.065	1
H(4)	0.39559	0.138282	0.177039	0.072	1
H(5)	0.462749	0.109884	0.334779	0.067	1
H(7A)	0.529814	0.132724	0.483754	0.128	1
H(7B)	0.625053	0.082745	0.563755	0.128	1
H(7C)	0.568084	0.013121	0.470191	0.128	1
H(9)	0.712199	0.011337	0.336097	0.047	1
H(10)	0.785421	-0.163722	0.379777	0.061	1
H(11)	0.935	-0.176058	0.505	0.062	1

H(12)	1.011907	-0.0106	0.582841	0.051	1
H(14A)	1.084179	0.15609	0.607964	0.068	1
H(14B)	1.036929	0.140173	0.672495	0.068	1
H(14C)	1.0618	0.26472	0.65216	0.068	1
H(16)	0.903758	0.191938	0.355006	0.047	1
H(17)	0.957288	0.277788	0.263197	0.059	1
H(18)	0.890943	0.446227	0.186711	0.066	1
H(19)	0.768986	0.528547	0.203119	0.059	1
H(21A)	0.617361	0.529201	0.179468	0.095	1
H(21B)	0.685171	0.611264	0.26113	0.095	1
H(21C)	0.591464	0.565705	0.25766	0.095	1
H(22A)	0.693118	0.429839	0.441031	0.044	0.608(3)
H(23A)	0.885308	0.453243	0.503519	0.05	0.608(3)
H(23B)	0.804574	0.536337	0.434892	0.05	0.608(3)
H(24A)	0.910367	0.643755	0.572484	0.054	0.608(3)
H(26A)	0.773814	0.554461	0.687925	0.064	0.608(3)
H(27A)	0.765662	0.365782	0.620193	0.055	0.608(3)
H(28A)	0.626285	0.432516	0.606419	0.102	0.608(3)
H(28B)	0.607532	0.355234	0.51969	0.102	0.608(3)
H(28C)	0.601301	0.490568	0.509906	0.102	0.608(3)
H(29A)	0.912059	0.45304	0.667598	0.079	0.608(3)
H(29B)	0.929175	0.577302	0.717175	0.079	0.608(3)
H(30A)	0.756452	0.777414	0.645397	0.113	0.608(3)
H(30B)	0.821318	0.812589	0.601537	0.113	0.608(3)
H(30C)	0.86274	0.737583	0.693257	0.113	0.608(3)
H(31A)	0.647163	0.701903	0.501719	0.082	0.608(3)
H(31B)	0.671819	0.601465	0.451829	0.082	0.608(3)
H(31C)	0.710813	0.726777	0.454441	0.082	0.608(3)
H(22B)	0.706084	0.43959	0.434923	0.044	0.392(3)
H(23C)	0.82811	0.528009	0.475553	0.05	0.392(3)
H(23D)	0.888361	0.4371	0.552935	0.05	0.392(3)
H(24B)	0.893718	0.627137	0.622736	0.054	0.392(3)
H(26B)	0.70235	0.521649	0.664691	0.064	0.392(3)
H(27B)	0.723885	0.337256	0.60263	0.055	0.392(3)
H(28D)	0.571443	0.391399	0.549963	0.102	0.392(3)
H(28E)	0.581784	0.333161	0.467907	0.102	0.392(3)
H(28F)	0.574348	0.468362	0.471649	0.102	0.392(3)
H(29C)	0.866389	0.426393	0.69269	0.079	0.392(3)
H(29D)	0.86664	0.546239	0.742844	0.079	0.392(3)
H(30D)	0.697334	0.746898	0.637237	0.113	0.392(3)
H(30E)	0.782245	0.789931	0.622876	0.113	0.392(3)
H(30F)	0.801419	0.711011	0.708889	0.113	0.392(3)
H(31D)	0.615177	0.668062	0.482216	0.082	0.392(3)
H(31E)	0.669021	0.593959	0.44144	0.082	0.392(3)
H(31F)	0.696136	0.7244	0.467612	0.082	0.392(3)
H(1A)	0.842(2)	0.280(3)	0.5323(19)	0.045(7)	1
H(1B)	0.745(2)	0.249(3)	0.5078(19)	0.044(7)	1

H(33)	0.285978	0.975697	0.172431	0.043	1
H(34)	0.220722	1.154725	0.129867	0.05	1
H(35)	0.076173	1.176734	-0.000547	0.051	1
H(36)	-0.005879	1.016621	-0.082794	0.047	1
H(38A)	-0.036086	0.861066	-0.171511	0.069	1
H(38B)	-0.086716	0.856059	-0.109355	0.069	1
H(38C)	-0.068973	0.741635	-0.150826	0.069	1
H(40)	0.093002	0.804813	0.147908	0.043	1
H(41)	0.038769	0.72314	0.241034	0.053	1
H(42)	0.105805	0.555708	0.320122	0.058	1
H(43)	0.222069	0.46754	0.300186	0.053	1
H(45A)	0.297129	0.375879	0.232186	0.094	1
H(45B)	0.366492	0.453167	0.316792	0.094	1
H(45C)	0.393276	0.417514	0.239042	0.094	1
H(47)	0.357356	0.739723	0.292078	0.048	1
H(48)	0.512697	0.787762	0.381586	0.063	1
H(49)	0.598322	0.854363	0.313406	0.07	1
H(50)	0.526278	0.879462	0.153524	0.06	1
H(52A)	0.361915	0.889173	-0.073708	0.125	1
H(52B)	0.45509	0.833745	0.005366	0.125	1
H(52C)	0.425968	0.959703	0.017619	0.125	1
H(53)	0.210199	0.527197	0.040523	0.041	1
H(54A)	0.360655	0.623472	0.03531	0.056	1
H(54B)	0.357682	0.509588	0.086579	0.056	1
H(55)	0.407443	0.462044	-0.026529	0.061	1
H(56A)	0.304672	0.513886	-0.183851	0.085	1
H(56B)	0.276755	0.617999	-0.136257	0.085	1
H(57)	0.158821	0.435495	-0.208334	0.059	1
H(58)	0.131823	0.605505	-0.138576	0.052	1
H(59A)	0.025651	0.456126	-0.167299	0.098	1
H(59B)	0.040356	0.527843	-0.079193	0.098	1
H(59C)	0.089851	0.407025	-0.066164	0.098	1
H(61A)	0.202798	0.259538	-0.044821	0.092	1
H(61B)	0.243618	0.361524	0.027606	0.092	1
H(61C)	0.311296	0.263604	0.027411	0.092	1
H(62A)	0.249055	0.240594	-0.167447	0.123	1
H(62B)	0.355972	0.250374	-0.091303	0.123	1
H(62C)	0.313958	0.335123	-0.177333	0.123	1
H(2A)	0.2356(18)	0.739(2)	-0.0175(18)	0.035(6)	1
H(2B)	0.147(2)	0.703(2)	-0.0233(19)	0.041(7)	1

Table S9. Anisotropic displacement parameters [Å^2] for **2a**

	U11	U22	U33	U23	U13	U12
O(1)	0.0438(9)	0.0698(12)	0.0530(10)	0.0141(9)	0.0275(8)	-0.0098(9)
O(2)	0.0293(7)	0.0379(8)	0.0490(9)	-0.0045(7)	0.0130(7)	0.0014(6)
O(3)	0.0577(10)	0.0353(8)	0.0517(10)	0.0109(7)	0.0323(8)	0.0094(7)
N(1)	0.0314(9)	0.0337(10)	0.0371(10)	-0.0017(8)	0.0162(8)	0.0055(8)
C(1)	0.0320(10)	0.0275(10)	0.0409(11)	0.0001(9)	0.0181(9)	0.0004(8)
C(2)	0.0389(12)	0.0374(12)	0.0418(12)	-0.0003(10)	0.0168(10)	-0.0002(9)
C(3)	0.0433(13)	0.0542(16)	0.0476(14)	0.0028(13)	0.0069(11)	-0.0015(12)
C(4)	0.0310(12)	0.0570(16)	0.0693(19)	0.0070(14)	0.0057(12)	-0.0074(12)
C(5)	0.0354(12)	0.0518(15)	0.078(2)	0.0116(14)	0.0258(13)	-0.0033(11)
C(6)	0.0350(11)	0.0398(13)	0.0506(13)	0.0066(10)	0.0212(10)	-0.0001(9)
C(7)	0.072(2)	0.115(3)	0.076(2)	0.031(2)	0.0412(18)	-0.019(2)
C(8)	0.0329(11)	0.0319(11)	0.0388(11)	-0.0002(9)	0.0221(9)	0.0016(9)
C(9)	0.0366(11)	0.0350(12)	0.0463(13)	-0.0036(10)	0.0200(10)	0.0016(9)
C(10)	0.0520(14)	0.0321(12)	0.0623(16)	-0.0069(12)	0.0231(13)	0.0003(11)
C(11)	0.0525(15)	0.0345(13)	0.0628(17)	0.0026(11)	0.0237(13)	0.0114(11)
C(12)	0.0370(11)	0.0422(13)	0.0469(13)	0.0015(11)	0.0182(10)	0.0089(10)
C(13)	0.0338(11)	0.0341(11)	0.0416(11)	-0.0014(9)	0.0221(9)	0.0029(9)
C(14)	0.0308(11)	0.0522(14)	0.0473(13)	-0.0007(11)	0.0147(10)	0.0004(10)
C(15)	0.0349(10)	0.0325(11)	0.0391(11)	-0.0048(9)	0.0197(9)	-0.0055(9)
C(16)	0.0380(11)	0.0414(12)	0.0441(12)	-0.0085(10)	0.0235(10)	-0.0084(9)
C(17)	0.0482(14)	0.0562(15)	0.0568(15)	-0.0100(13)	0.0356(13)	-0.0120(12)
C(18)	0.0687(17)	0.0586(16)	0.0553(15)	-0.0062(13)	0.0432(15)	-0.0204(14)
C(19)	0.0674(17)	0.0396(13)	0.0497(14)	0.0012(11)	0.0351(13)	-0.0069(12)
C(20)	0.0487(13)	0.0328(11)	0.0441(12)	-0.0040(10)	0.0267(11)	-0.0077(10)
C(21)	0.091(2)	0.0526(16)	0.0530(16)	0.0183(13)	0.0403(16)	0.0278(15)
C(22A)	0.0388(11)	0.0354(11)	0.0361(11)	-0.0027(9)	0.0176(9)	0.0077(9)
C(23A)	0.0500(15)	0.0365(18)	0.048(3)	-0.005(2)	0.031(3)	-0.0061(13)
C(24A)	0.044(2)	0.0378(18)	0.045(2)	-0.0042(17)	0.0136(15)	-0.0008(15)
C(25A)	0.071(4)	0.028(2)	0.0381(17)	-0.0008(17)	0.028(2)	0.004(2)
C(26A)	0.088(4)	0.043(2)	0.0380(17)	-0.0012(16)	0.037(3)	0.011(2)
C(27A)	0.068(5)	0.035(3)	0.049(3)	0.009(2)	0.040(4)	0.012(3)
C(28A)	0.082(5)	0.056(4)	0.105(6)	0.004(3)	0.076(5)	0.011(3)
C(29A)	0.074(3)	0.053(2)	0.040(2)	0.0000(18)	0.003(2)	0.010(2)
C(30A)	0.108(5)	0.049(3)	0.067(2)	-0.017(2)	0.040(3)	0.004(3)
C(31A)	0.052(8)	0.041(6)	0.061(3)	0.009(3)	0.019(5)	0.012(5)
C(22B)	0.0388(11)	0.0354(11)	0.0361(11)	-0.0027(9)	0.0176(9)	0.0077(9)
C(23B)	0.0500(15)	0.0365(18)	0.048(3)	-0.005(2)	0.031(3)	-0.0061(13)
C(24B)	0.044(2)	0.0378(18)	0.045(2)	-0.0042(17)	0.0136(15)	-0.0008(15)
C(25B)	0.071(4)	0.028(2)	0.0381(17)	-0.0008(17)	0.028(2)	0.004(2)
C(26B)	0.088(4)	0.043(2)	0.0380(17)	-0.0012(16)	0.037(3)	0.011(2)
C(27B)	0.068(5)	0.035(3)	0.049(3)	0.009(2)	0.040(4)	0.012(3)
C(28B)	0.082(5)	0.056(4)	0.105(6)	0.004(3)	0.076(5)	0.011(3)

C(29B)	0.074(3)	0.053(2)	0.040(2)	0.0000(18)	0.003(2)	0.010(2)
C(30B)	0.108(5)	0.049(3)	0.067(2)	-0.017(2)	0.040(3)	0.004(3)
C(31B)	0.052(8)	0.041(6)	0.061(3)	0.009(3)	0.019(5)	0.012(5)
B(2)	0.0308(11)	0.0282(11)	0.0373(12)	-0.0022(10)	0.0186(10)	-0.0005(9)
O(4)	0.0274(7)	0.0328(8)	0.0483(9)	-0.0007(7)	0.0082(7)	0.0010(6)
O(5)	0.0543(10)	0.0342(8)	0.0468(9)	0.0119(7)	0.0273(8)	0.0137(7)
O(6)	0.0408(8)	0.0574(11)	0.0472(9)	0.0052(8)	0.0260(8)	-0.0080(8)
N(2)	0.0297(9)	0.0322(9)	0.0350(9)	0.0004(8)	0.0160(8)	0.0020(7)
C(32)	0.0281(10)	0.0306(11)	0.0352(11)	0.0015(8)	0.0186(9)	-0.0001(8)
C(33)	0.0322(10)	0.0342(11)	0.0410(11)	0.0003(9)	0.0183(9)	-0.0018(9)
C(34)	0.0417(12)	0.0312(11)	0.0542(14)	-0.0027(10)	0.0237(11)	-0.0025(10)
C(35)	0.0461(13)	0.0286(11)	0.0574(15)	0.0069(10)	0.0287(12)	0.0076(10)
C(36)	0.0356(11)	0.0374(12)	0.0439(12)	0.0062(10)	0.0193(10)	0.0078(9)
C(37)	0.0314(10)	0.0315(11)	0.0395(11)	0.0019(9)	0.0190(9)	-0.0002(9)
C(38)	0.0333(11)	0.0476(13)	0.0446(13)	0.0032(11)	0.0084(10)	-0.0022(10)
C(39)	0.0341(10)	0.0284(10)	0.0327(10)	-0.0026(8)	0.0162(9)	-0.0041(8)
C(40)	0.0355(11)	0.0339(11)	0.0430(12)	-0.0019(9)	0.0222(10)	-0.0033(9)
C(41)	0.0498(13)	0.0445(13)	0.0507(13)	-0.0080(11)	0.0337(12)	-0.0103(11)
C(42)	0.0622(15)	0.0484(14)	0.0473(13)	-0.0033(11)	0.0361(12)	-0.0158(12)
C(43)	0.0604(15)	0.0333(11)	0.0386(12)	0.0029(10)	0.0227(11)	-0.0102(10)
C(44)	0.0420(11)	0.0315(11)	0.0356(11)	-0.0015(9)	0.0195(9)	-0.0007(9)
C(45)	0.086(2)	0.0504(16)	0.0543(16)	0.0189(13)	0.0358(16)	0.0306(15)
C(46)	0.0301(10)	0.0275(10)	0.0396(11)	0.0010(9)	0.0159(9)	0.0035(8)
C(47)	0.0353(11)	0.0398(12)	0.0403(12)	0.0022(10)	0.0133(10)	0.0028(9)
C(48)	0.0404(13)	0.0549(16)	0.0445(13)	0.0007(12)	0.0053(11)	0.0033(12)
C(49)	0.0290(12)	0.0575(17)	0.0703(18)	-0.0008(14)	0.0080(12)	-0.0026(11)
C(50)	0.0335(12)	0.0492(14)	0.0674(17)	0.0019(12)	0.0243(12)	-0.0044(11)
C(51)	0.0313(10)	0.0342(11)	0.0485(13)	0.0004(10)	0.0187(10)	0.0013(9)
C(52)	0.073(2)	0.117(3)	0.074(2)	0.017(2)	0.0467(19)	-0.022(2)
C(53)	0.0330(10)	0.0338(11)	0.0357(11)	-0.0002(9)	0.0154(9)	0.0049(9)
C(54)	0.0333(11)	0.0504(13)	0.0531(13)	-0.0095(11)	0.0180(10)	0.0063(10)
C(55)	0.0485(13)	0.0442(13)	0.0766(17)	-0.0146(12)	0.0438(13)	-0.0066(10)
C(56)	0.112(3)	0.0631(18)	0.0701(19)	-0.0094(15)	0.070(2)	-0.0224(17)
C(57)	0.0702(17)	0.0427(13)	0.0375(12)	-0.0057(11)	0.0273(12)	-0.0066(12)
C(58)	0.0442(12)	0.0361(11)	0.0389(11)	-0.0004(9)	0.0112(10)	0.0070(9)
C(59)	0.0345(12)	0.0694(18)	0.0764(19)	-0.0134(15)	0.0144(13)	0.0010(12)
C(60)	0.0446(13)	0.0381(12)	0.0704(16)	-0.0092(11)	0.0360(13)	-0.0002(10)
C(61)	0.0545(15)	0.0405(13)	0.088(2)	0.0198(14)	0.0333(15)	0.0097(12)
C(62)	0.079(2)	0.0618(19)	0.128(3)	-0.048(2)	0.067(2)	-0.0136(16)
B(1)	0.0284(11)	0.0295(12)	0.0328(12)	0.0010(9)	0.0150(10)	0.0018(9)

Table S10. Crystal data and structure refinement for **2e**.

CCDC-Nr	2205402
Empirical formula	C29 H32 B N O3
Formula weight [g/mol]	453.36
Crystal system	Orthorhombic
Space group	P212121 (19)
Lattice parameters [Å]	
a	8.08500(7)
b	16.78762(14)
c	18.11408(16)
α	90
β	90
γ	90
Density [g/cm^3]	1.225
Crystal size [mm^3]	0.361 x 0.064 x 0.048
Volume [Å^3]	2458.58(4)
Z	4
Temperature [K]	169.99(10)
Diffraction Device	XtaLAB Synergy, Dualflex, HyPix
Radiation Type	1.54184 Å (Cu K/ micro-focus sealed X-ray tube)
F(000)	968
Absorption coefficient [mm^{-1}]	0.611
Absorption correction	Semi-empirical from equivalents
Measurement range	3.6 - 66.5
Index range	-9 < h < 9
	-19 < k < 16
	-21 < l < 21
Measured reflexes	27801
Independent	4340
Observed	4134
R(int)	0.0505
Completeness (%) / theta (°)	100.0 / 66.489
Transmission (min / max)	0.57269 / 1.00000
R1 (observed/all)	0.0292 / 0.0312
wR2 (observed/all)	0.0731 / 0.0744
GooF = S	1.041
Rest electron density max./min. [e-/Å^3]	-0.172 / 0.245

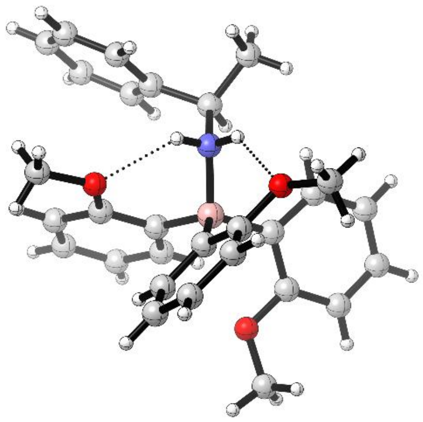

Table S11. Atomic coordinates and equivalent isotropic displacement parameters [Å^2] for **2e**

	**x**	**y**	**z**	**U(eq)**
O(1)	1.03211(16)	0.53500(8)	0.49895(7)	0.0301(3)
B(1)	0.7316(2)	0.52164(12)	0.58064(11)	0.0212(4)
N(1)	0.57783(19)	0.48866(9)	0.63248(8)	0.0221(3)
C(2)	0.8962(2)	0.46690(10)	0.59543(9)	0.0219(3)
O(2)	0.59467(18)	0.37553(7)	0.52014(7)	0.0297(3)
C(3)	0.9070(2)	0.40604(11)	0.64815(10)	0.0275(4)
O(3)	0.47166(15)	0.63804(7)	0.57556(7)	0.0259(3)
C(4)	1.0470(3)	0.35862(11)	0.65818(11)	0.0321(4)
C(5)	1.1825(2)	0.36994(11)	0.61352(12)	0.0310(4)
C(6)	1.1783(2)	0.42810(11)	0.55935(11)	0.0290(4)
C(8)	1.1538(3)	0.53357(13)	0.44234(11)	0.0360(4)
C(7)	1.0384(2)	0.47570(10)	0.55100(10)	0.0238(4)
C(9)	0.6653(2)	0.51032(10)	0.49587(9)	0.0216(3)
C(10)	0.6705(2)	0.57194(11)	0.44438(10)	0.0261(4)
C(11)	0.6131(2)	0.56394(12)	0.37234(10)	0.0307(4)
C(12)	0.5467(2)	0.49249(12)	0.34955(11)	0.0331(4)
C(13)	0.5401(2)	0.42879(12)	0.39831(11)	0.0314(4)
C(14)	0.6008(2)	0.43793(11)	0.46968(10)	0.0240(4)
C(15)	0.5835(5)	0.29771(14)	0.49225(15)	0.0686(9)
C(16)	0.7528(2)	0.61477(10)	0.60366(10)	0.0211(4)
C(17)	0.9013(2)	0.64604(10)	0.62967(10)	0.0252(4)
C(18)	0.9201(2)	0.72578(11)	0.65013(11)	0.0303(4)
C(19)	0.7870(2)	0.77679(11)	0.64516(11)	0.0309(4)
C(20)	0.6356(2)	0.74875(10)	0.61990(10)	0.0271(4)
C(21)	0.6200(2)	0.66930(10)	0.59960(9)	0.0220(4)
C(22)	0.3380(2)	0.69179(12)	0.56326(12)	0.0341(4)
C(23)	0.5877(2)	0.49550(10)	0.71562(9)	0.0243(4)
C(24)	0.5211(3)	0.41973(11)	0.75161(11)	0.0324(4)
C(25)	0.4959(2)	0.56794(11)	0.74448(10)	0.0247(4)
C(26)	0.3270(2)	0.57759(12)	0.73290(10)	0.0281(4)
C(27)	0.2440(3)	0.64185(12)	0.76366(11)	0.0327(4)
C(28)	0.3273(3)	0.69635(13)	0.80692(12)	0.0391(5)
C(29)	0.4952(3)	0.68679(13)	0.81851(14)	0.0453(5)
C(30)	0.5795(3)	0.62323(13)	0.78746(12)	0.0358(5)
H(2)	0.491(3)	0.5164(14)	0.6195(13)	0.030(5)
H(1)	0.557(3)	0.4386(15)	0.6191(13)	0.034(6)
H(3)	0.81376	0.396516	0.678809	0.033
H(4)	1.048672	0.318892	0.695521	0.039
H(5)	1.27853	0.33802	0.619789	0.037
H(6)	1.270914	0.435557	0.527832	0.035
H(8A)	1.152475	0.481503	0.417887	0.054
H(8B)	1.26322	0.542876	0.464003	0.054
H(8C)	1.129752	0.575345	0.406116	0.054
H(10)	0.715342	0.62176	0.459155	0.031
H(11)	0.619613	0.60755	0.339038	0.037

H(12)	0.505742	0.486825	0.300682	0.04
H(13)	0.494411	0.37931	0.383066	0.038
H(15A)	0.473972	0.289578	0.470354	0.103
H(15B)	0.600116	0.259476	0.532459	0.103
H(15C)	0.668685	0.289734	0.45447	0.103
H(17)	0.99411	0.611583	0.633671	0.03
H(18)	1.023902	0.744706	0.667343	0.036
H(19)	0.798836	0.831078	0.659001	0.037
H(20)	0.543418	0.783635	0.616532	0.033
H(22A)	0.369992	0.730956	0.525756	0.051
H(22B)	0.311166	0.719342	0.609422	0.051
H(22C)	0.241054	0.662048	0.546125	0.051
H(23)	0.7068	0.501061	0.729565	0.029
H(24A)	0.402261	0.415171	0.741829	0.049
H(24B)	0.539857	0.422069	0.805017	0.049
H(24C)	0.578463	0.373318	0.731078	0.049
H(26)	0.268253	0.539988	0.703781	0.034
H(27)	0.128921	0.648379	0.754872	0.039
H(28)	0.269916	0.739913	0.82849	0.047
H(29)	0.553327	0.724213	0.848069	0.054
H(30)	0.694919	0.617487	0.795627	0.043

Table S12. Anisotropic displacement parameters [Å^2] for **2e**

	**U11**	**U22**	**U33**	**U23**	**U13**	**U12**
O(1)	0.0234(6)	0.0315(7)	0.0354(7)	0.0098(6)	0.0060(5)	0.0033(5)
B(1)	0.0184(9)	0.0212(9)	0.0239(9)	-0.0001(8)	0.0010(7)	-0.0002(7)
N(1)	0.0215(7)	0.0214(7)	0.0234(7)	-0.0030(6)	0.0005(6)	-0.0014(6)
C(2)	0.0213(8)	0.0187(8)	0.0256(8)	-0.0028(6)	-0.0031(7)	-0.0002(7)
O(2)	0.0411(7)	0.0213(6)	0.0267(6)	-0.0035(5)	0.0023(6)	-0.0033(5)
C(3)	0.0289(9)	0.0241(8)	0.0293(9)	0.0015(7)	0.0023(8)	0.0025(7)
O(3)	0.0179(6)	0.0259(6)	0.0339(6)	-0.0024(5)	-0.0034(5)	0.0023(5)
C(4)	0.0385(11)	0.0233(9)	0.0346(10)	0.0038(7)	-0.0058(8)	0.0047(8)
C(5)	0.0268(9)	0.0249(9)	0.0412(11)	-0.0033(8)	-0.0074(8)	0.0069(7)
C(6)	0.0228(9)	0.0287(9)	0.0354(10)	-0.0033(8)	-0.0001(8)	0.0022(8)
C(8)	0.0332(10)	0.0419(11)	0.0330(10)	0.0067(9)	0.0067(9)	0.0011(9)
C(7)	0.0227(8)	0.0212(8)	0.0276(8)	-0.0009(7)	-0.0032(7)	0.0005(7)
C(9)	0.0148(8)	0.0250(8)	0.0251(8)	-0.0019(7)	0.0025(7)	0.0028(7)
C(10)	0.0250(9)	0.0252(9)	0.0282(9)	-0.0002(7)	0.0032(7)	0.0012(8)
C(11)	0.0300(10)	0.0356(10)	0.0266(9)	0.0059(8)	0.0028(8)	0.0076(8)
C(12)	0.0302(10)	0.0455(12)	0.0236(9)	-0.0040(8)	-0.0014(8)	0.0043(8)
C(13)	0.0303(9)	0.0354(10)	0.0284(9)	-0.0085(8)	0.0008(8)	-0.0017(8)
C(14)	0.0213(8)	0.0258(9)	0.0248(8)	-0.0021(7)	0.0037(7)	0.0020(7)
C(15)	0.134(3)	0.0272(11)	0.0450(13)	-0.0043(10)	0.0091(17)	-0.0035(15)
C(16)	0.0207(8)	0.0226(8)	0.0201(8)	0.0005(6)	0.0005(7)	0.0002(7)
C(17)	0.0207(8)	0.0241(8)	0.0307(9)	-0.0007(7)	-0.0018(7)	0.0016(7)
C(18)	0.0247(9)	0.0270(9)	0.0393(10)	-0.0042(8)	-0.0067(8)	-0.0023(7)

C(19)	0.0333(10)	0.0198(9)	0.0395(11)	-0.0046(8)	-0.0042(9)	0.0006(7)
C(20)	0.0247(9)	0.0227(8)	0.0339(10)	-0.0010(7)	-0.0007(7)	0.0050(7)
C(21)	0.0195(8)	0.0241(8)	0.0225(8)	0.0003(6)	0.0004(7)	-0.0004(7)
C(22)	0.0220(9)	0.0358(10)	0.0445(11)	-0.0017(9)	-0.0053(9)	0.0079(8)
C(23)	0.0245(9)	0.0260(9)	0.0224(8)	-0.0018(7)	-0.0004(7)	-0.0004(7)
C(24)	0.0379(10)	0.0286(10)	0.0307(9)	0.0025(8)	0.0065(9)	-0.0011(8)
C(25)	0.0269(9)	0.0256(8)	0.0216(8)	0.0009(7)	0.0028(7)	-0.0015(7)
C(26)	0.0287(9)	0.0324(10)	0.0232(8)	-0.0019(7)	0.0002(7)	-0.0012(8)
C(27)	0.0304(10)	0.0382(11)	0.0296(10)	0.0049(8)	0.0035(8)	0.0056(9)
C(28)	0.0472(12)	0.0297(10)	0.0405(11)	-0.0048(9)	0.0104(10)	0.0080(9)
C(29)	0.0456(13)	0.0372(11)	0.0530(13)	-0.0206(10)	-0.0012(11)	-0.0061(10)
C(30)	0.0286(10)	0.0358(11)	0.0429(11)	-0.0111(9)	-0.0027(9)	-0.0021(8)

Table S13. Crystal data and structure refinement for **2f**.

CCDC-Nr	2205403
Empirical formula	C30 H34 B N O4
Formula weight [g/mol]	483.39
Crystal system	Orthorhombic
Space group	P212121 (19)
Lattice parameters [Å]	
a	7.75206(8)
b	14.16601(17)
c	23.4503(3)
α	90
β	90
γ	90
Density [g/cm^3]	1.247
Crystal size [mm^3]	0.191 x 0.044 x 0.040
Volume [$Å^3$]	2575.21(5)
Z	4
Temperature [K]	170.00(10)
Diffraction Device	XtaLAB Synergy, Dualflex, HyPix
Radiation Type	1.54184 Å (Cu K/ micro-focus sealed X-ray tube)
F(000)	1032
Absorption coefficient [mm^{-1}]	0.645
Absorption correction	Semi-empirical from equivalents
Measurement range	3.6 - 66.4
Index range	-9 < h < 8
	-16 < k < 16
	-27 < l < 27
Measured reflexes	26431
Independent	4536
Observed	4245
R(int)	0.0428
Completeness (%) / theta (°)	100.0 / 66.420
Transmission (min / max)	0.52401 / 1.00000
R1 (observed/all)	0.0325 / 0.0359
wR2 (observed/all)	0.0863 / 0.0888
GooF = S	1.051
Rest electron density max./min. [$e^-/Å^3$]	-0.126 / 0.492

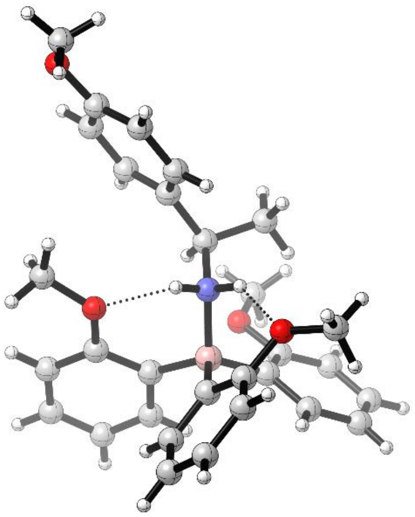

**Table S14.** Atomic coordinates and equivalent isotropic displacement parameters [Å^2] for **2f**				
	x	y	z	U(eq)
O(1)	0.4162(2)	0.62610(12)	0.29109(7)	0.0412(4)
O(2)	0.9602(2)	0.43137(11)	0.33348(7)	0.0387(4)
O(4)	0.6685(2)	0.60341(12)	0.47470(6)	0.0380(4)
O(5)	0.1839(2)	0.34824(15)	0.58165(8)	0.0523(5)
N(2)	0.5859(2)	0.52941(13)	0.37298(8)	0.0290(4)
C(1)	0.8002(3)	0.61458(18)	0.24059(9)	0.0343(5)
C(2)	0.8634(3)	0.5867(2)	0.18732(10)	0.0422(6)
C(3)	0.9549(3)	0.5041(2)	0.18308(10)	0.0451(6)
C(4)	0.9865(3)	0.45082(18)	0.23120(10)	0.0403(5)
C(5)	0.9261(3)	0.48088(16)	0.28420(9)	0.0333(5)
C(6)	0.6413(3)	0.70134(15)	0.34200(8)	0.0279(4)
C(7)	0.8830(3)	0.61218(14)	0.40284(8)	0.0285(4)
C(8)	0.8255(3)	0.56304(16)	0.29086(9)	0.0303(4)
C(9)	0.3034(3)	0.6304(2)	0.24317(11)	0.0492(6)
C(10)	1.0553(3)	0.63103(15)	0.38990(9)	0.0320(4)
C(11)	1.1781(3)	0.65554(16)	0.43075(10)	0.0357(5)
C(12)	1.1299(3)	0.66003(17)	0.48752(10)	0.0382(5)
C(13)	0.9615(3)	0.64103(16)	0.50287(9)	0.0361(5)
C(14)	0.8400(3)	0.61845(15)	0.46137(9)	0.0304(4)
C(15)	0.0067(4)	0.3739(2)	0.58189(13)	0.0591(7)
C(16)	0.7101(3)	0.78635(16)	0.36179(9)	0.0309(4)
C(17)	0.6299(3)	0.87340(17)	0.35356(10)	0.0386(5)
C(18)	0.4725(3)	0.87746(18)	0.32559(11)	0.0422(6)
C(19)	0.3990(3)	0.79484(18)	0.30525(10)	0.0401(5)
C(20)	0.4841(3)	0.70957(17)	0.31244(9)	0.0336(5)
C(21)	0.6215(4)	0.59241(19)	0.53319(10)	0.0436(6)
C(22)	0.3183(3)	0.42670(17)	0.43860(10)	0.0363(5)
C(23)	0.2090(3)	0.40870(18)	0.48461(11)	0.0403(5)
C(24)	0.2760(3)	0.36692(17)	0.53309(10)	0.0381(5)
C(25)	0.4492(3)	0.34123(19)	0.53458(11)	0.0431(6)
C(26)	0.5550(3)	0.36079(17)	0.48870(10)	0.0384(5)
C(27R)	0.6114(3)	0.42872(15)	0.39083(9)	0.0319(5)
C(28)	1.0344(4)	0.3404(2)	0.32824(13)	0.0524(7)
C(30)	0.5797(4)	0.36385(19)	0.34014(11)	0.0464(6)
C(31)	0.4921(3)	0.40494(15)	0.44031(9)	0.0321(5)
B(1)	0.7426(3)	0.60032(17)	0.35151(10)	0.0275(5)
HN2	0.504(4)	0.534(2)	0.3438(12)	0.041(7)
HN1	0.550(4)	0.558(2)	0.4035(13)	0.040(7)
H(1)	0.73644	0.671738	0.242802	0.041
H(2)	0.84329	0.624508	0.154517	0.051
H(3)	0.996371	0.483551	0.14703	0.054
H(4)	1.049603	0.393537	0.22823	0.048
H(9A)	0.287881	0.566908	0.227402	0.074

H(9B)	0.353336	0.671728	0.214004	0.074
H(9C)	0.191323	0.655561	0.255155	0.074
H(10)	1.090867	0.626948	0.351207	0.038
H(11)	1.293499	0.668982	0.419761	0.043
H(12)	1.212373	0.676153	0.515845	0.046
H(13)	0.928471	0.643379	0.541858	0.043
H(15A)	-0.041825	0.36262	0.619865	0.089
H(15B)	-0.055585	0.335797	0.553729	0.089
H(15C)	-0.004573	0.440895	0.572223	0.089
H(16)	0.816605	0.7848	0.381819	0.037
H(17)	0.682876	0.929621	0.367031	0.046
H(18)	0.415591	0.936244	0.320418	0.051
H(19)	0.2905	0.796631	0.286423	0.048
H(21A)	0.499933	0.573857	0.535733	0.065
H(21B)	0.638633	0.65231	0.553307	0.065
H(21C)	0.693581	0.543503	0.550669	0.065
H(22)	0.272014	0.454723	0.405137	0.044
H(23)	0.090256	0.424916	0.482647	0.048
H(25)	0.494658	0.310189	0.56719	0.052
H(26)	0.673389	0.343613	0.490384	0.046
H(27)	0.733412	0.420566	0.403719	0.038
H(28A)	1.043339	0.311262	0.366036	0.079
H(28B)	1.149636	0.345841	0.311391	0.079
H(28C)	0.961513	0.301104	0.303683	0.079
H(30A)	0.607795	0.298749	0.350792	0.07
H(30B)	0.652762	0.383459	0.308159	0.07
H(30C)	0.458185	0.367595	0.328847	0.07

Table S15. Anisotropic displacement parameters [Å^2] for **2f**

	U11	U22	U33	U23	U13	U12
O(1)	0.0393(9)	0.0425(9)	0.0417(9)	0.0102(7)	-0.0146(7)	-0.0071(7)
O(2)	0.0423(9)	0.0352(8)	0.0388(8)	-0.0032(7)	0.0010(7)	0.0082(7)
O(4)	0.0383(8)	0.0497(10)	0.0261(7)	-0.0018(7)	0.0054(6)	-0.0025(7)
O(5)	0.0507(10)	0.0655(12)	0.0406(9)	0.0110(9)	0.0112(8)	-0.0020(9)
N(2)	0.0314(9)	0.0298(9)	0.0258(9)	0.0025(7)	-0.0001(8)	-0.0008(7)
C(1)	0.0314(11)	0.0436(13)	0.0279(11)	-0.0006(9)	-0.0002(8)	-0.0069(9)
C(2)	0.0337(11)	0.0658(16)	0.0271(11)	0.0002(10)	-0.0007(9)	-0.0114(11)
C(3)	0.0362(12)	0.0686(17)	0.0306(12)	-0.0164(11)	0.0046(9)	-0.0109(12)
C(4)	0.0298(11)	0.0473(13)	0.0439(13)	-0.0166(10)	0.0041(10)	-0.0044(10)
C(5)	0.0286(10)	0.0383(11)	0.0331(11)	-0.0053(9)	-0.0005(9)	-0.0055(9)
C(6)	0.0272(10)	0.0330(11)	0.0237(9)	0.0034(8)	0.0042(8)	0.0007(8)
C(7)	0.0324(10)	0.0259(10)	0.0273(10)	-0.0002(8)	-0.0002(8)	0.0031(8)
C(8)	0.0274(10)	0.0361(11)	0.0274(10)	-0.0030(8)	-0.0002(8)	-0.0058(8)
C(9)	0.0453(14)	0.0614(16)	0.0409(14)	0.0129(12)	-0.0150(11)	-0.0119(13)
C(10)	0.0340(11)	0.0325(10)	0.0296(10)	-0.0024(8)	-0.0014(8)	0.0032(9)
C(11)	0.0293(10)	0.0363(12)	0.0417(12)	-0.0057(10)	-0.0031(9)	0.0030(9)
C(12)	0.0418(12)	0.0365(12)	0.0363(12)	-0.0061(10)	-0.0114(10)	0.0038(10)
C(13)	0.0463(13)	0.0356(11)	0.0265(10)	-0.0026(9)	-0.0045(9)	0.0017(10)
C(14)	0.0347(11)	0.0276(10)	0.0291(10)	-0.0001(8)	0.0001(8)	0.0025(8)
C(15)	0.0526(16)	0.0681(18)	0.0565(17)	0.0099(15)	0.0194(14)	0.0038(14)
C(16)	0.0333(10)	0.0326(11)	0.0270(10)	0.0013(8)	0.0023(8)	0.0010(9)
C(17)	0.0463(13)	0.0331(12)	0.0364(11)	0.0022(10)	0.0073(10)	0.0024(10)
C(18)	0.0462(13)	0.0382(12)	0.0422(13)	0.0102(10)	0.0065(10)	0.0132(11)
C(19)	0.0344(11)	0.0464(13)	0.0397(12)	0.0115(11)	0.0004(10)	0.0075(10)
C(20)	0.0314(10)	0.0403(12)	0.0289(10)	0.0085(9)	0.0013(9)	-0.0018(9)
C(21)	0.0547(15)	0.0460(14)	0.0302(11)	-0.0018(10)	0.0107(11)	-0.0049(11)
C(22)	0.0411(12)	0.0343(12)	0.0336(11)	0.0070(9)	-0.0016(9)	0.0023(10)
C(23)	0.0384(13)	0.0389(13)	0.0435(13)	0.0056(10)	0.0031(10)	0.0028(10)
C(24)	0.0439(13)	0.0371(12)	0.0334(12)	0.0054(9)	0.0053(9)	-0.0048(10)
C(25)	0.0491(14)	0.0470(13)	0.0331(12)	0.0117(10)	-0.0045(10)	-0.0023(11)
C(26)	0.0364(12)	0.0398(12)	0.0391(12)	0.0050(10)	-0.0033(9)	0.0018(10)
C(27R)	0.0360(11)	0.0281(10)	0.0318(11)	0.0010(8)	-0.0004(9)	0.0010(8)
C(28)	0.0522(15)	0.0413(14)	0.0635(17)	-0.0067(12)	-0.0054(13)	0.0110(12)
C(30)	0.0557(15)	0.0420(13)	0.0414(13)	-0.0098(11)	0.0098(11)	-0.0091(12)
C(31)	0.0381(11)	0.0255(10)	0.0328(11)	0.0001(8)	0.0010(9)	-0.0001(9)
B(1)	0.0276(11)	0.0291(12)	0.0257(11)	0.0007(9)	-0.0001(9)	-0.0020(9)

5. NMR spectra

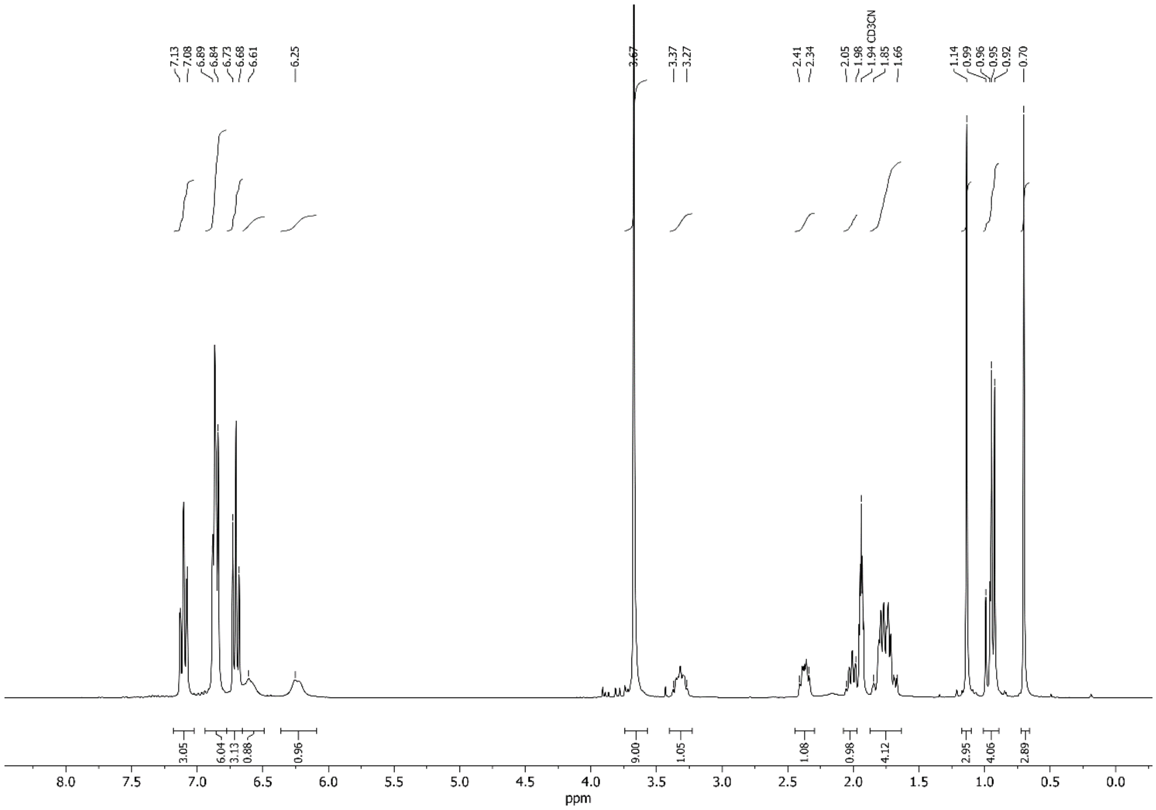

Fig. S7. 300 MHz ^{1}H-NMR of **2a** in ACN-*d3*

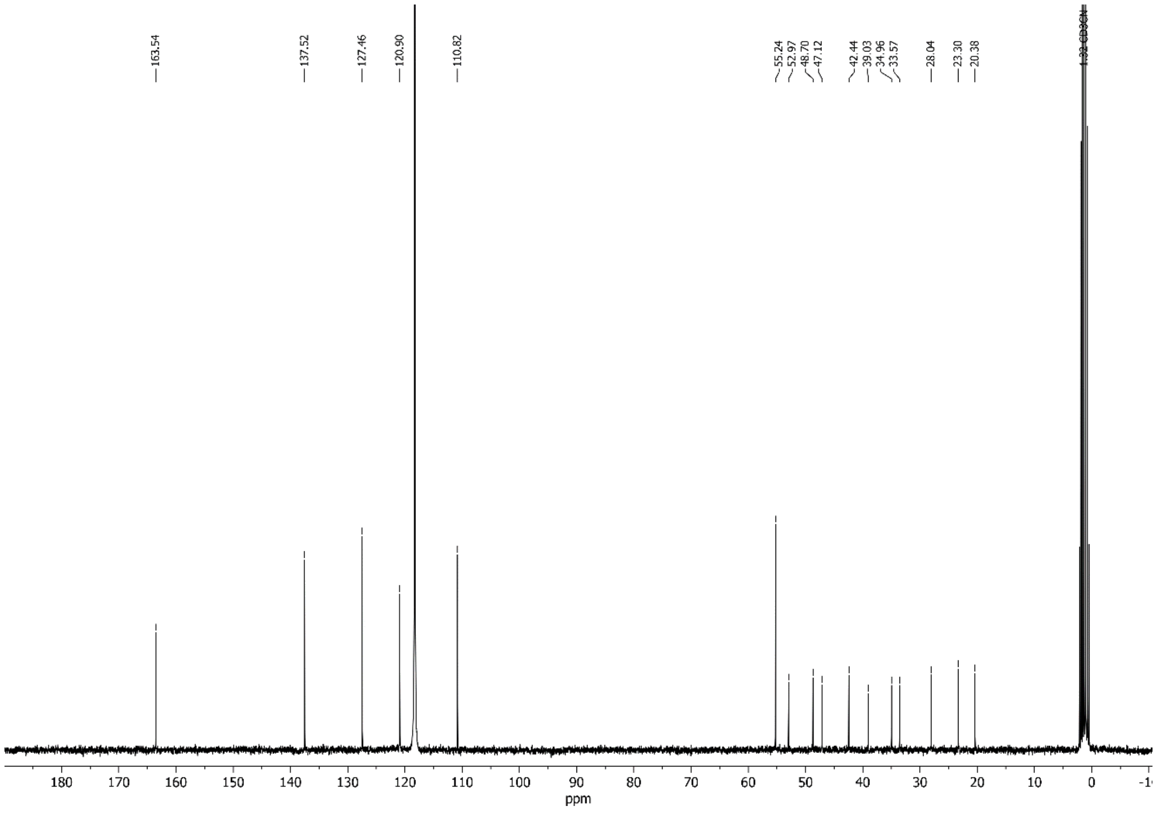

Fig. S8. 75 MHz ^{13}C-NMR of **2a** in ACN-*d3*

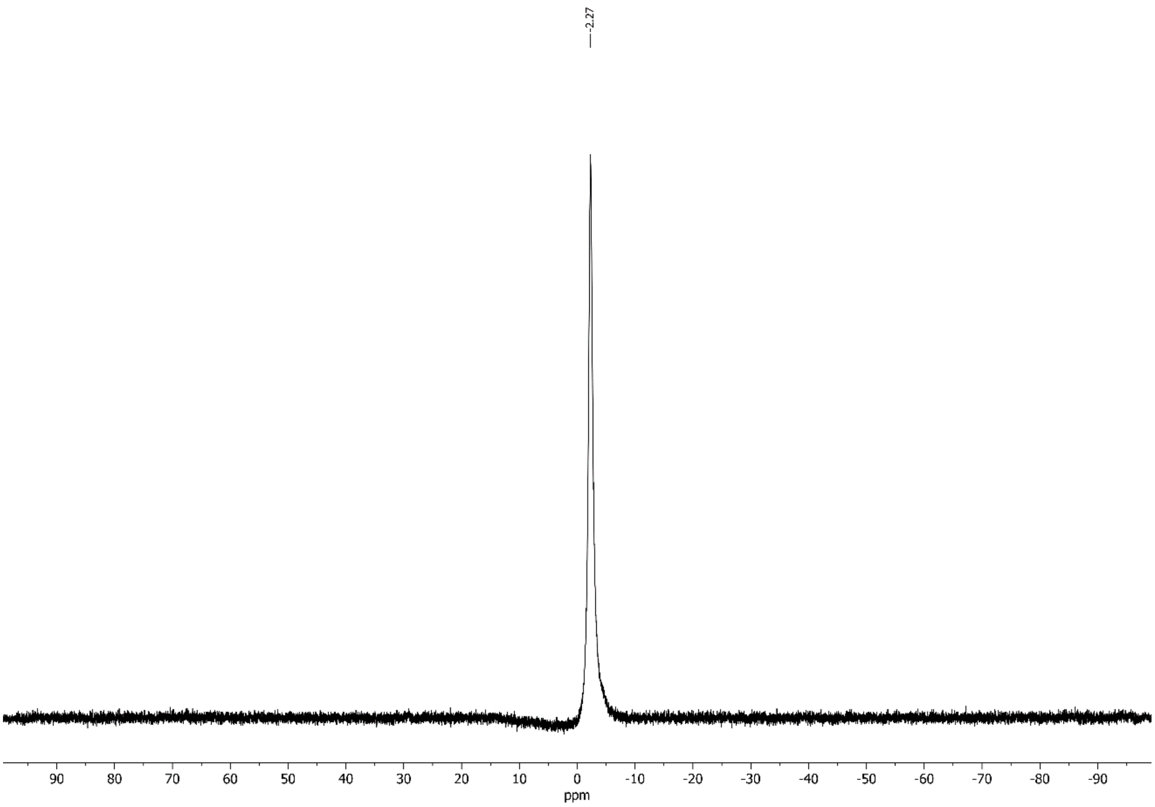

Fig. S9. 128 MHz ^{11}B-NMR of **2a** in ACN-*d3*

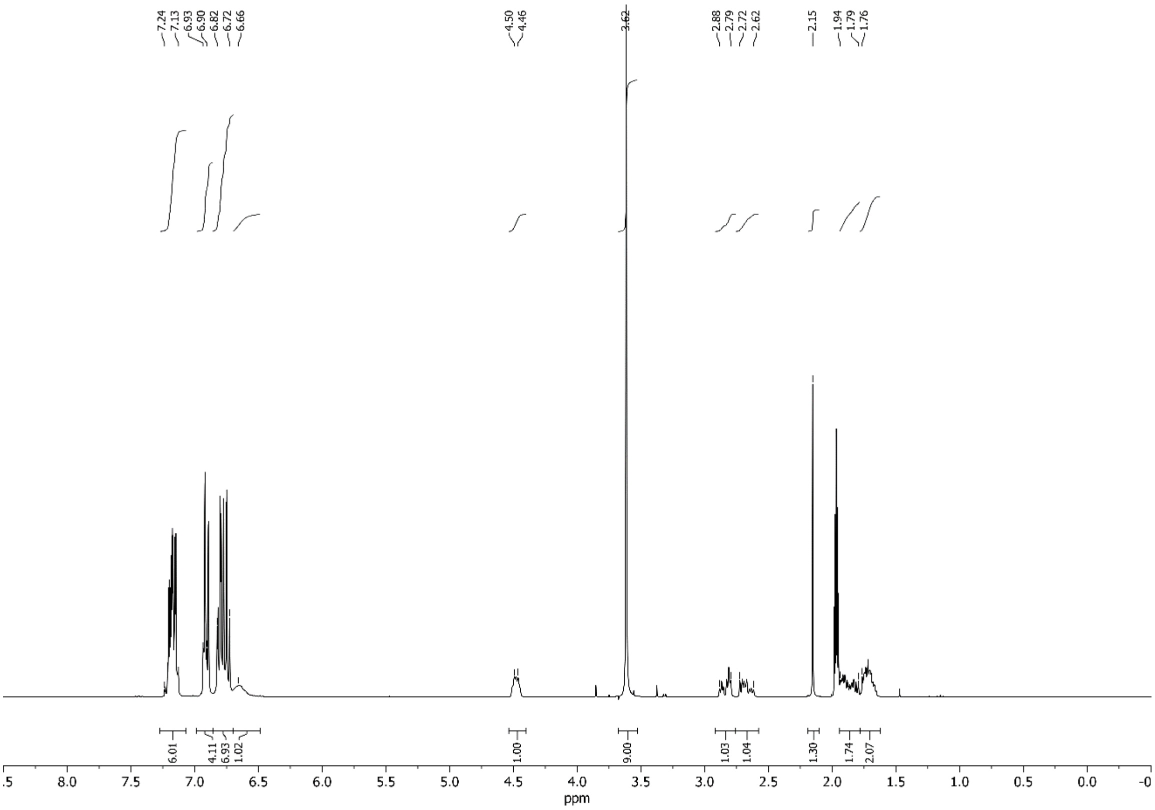

Fig. S10. 300 MHz ^{1}H-NMR of **2b** in ACN-*d3*

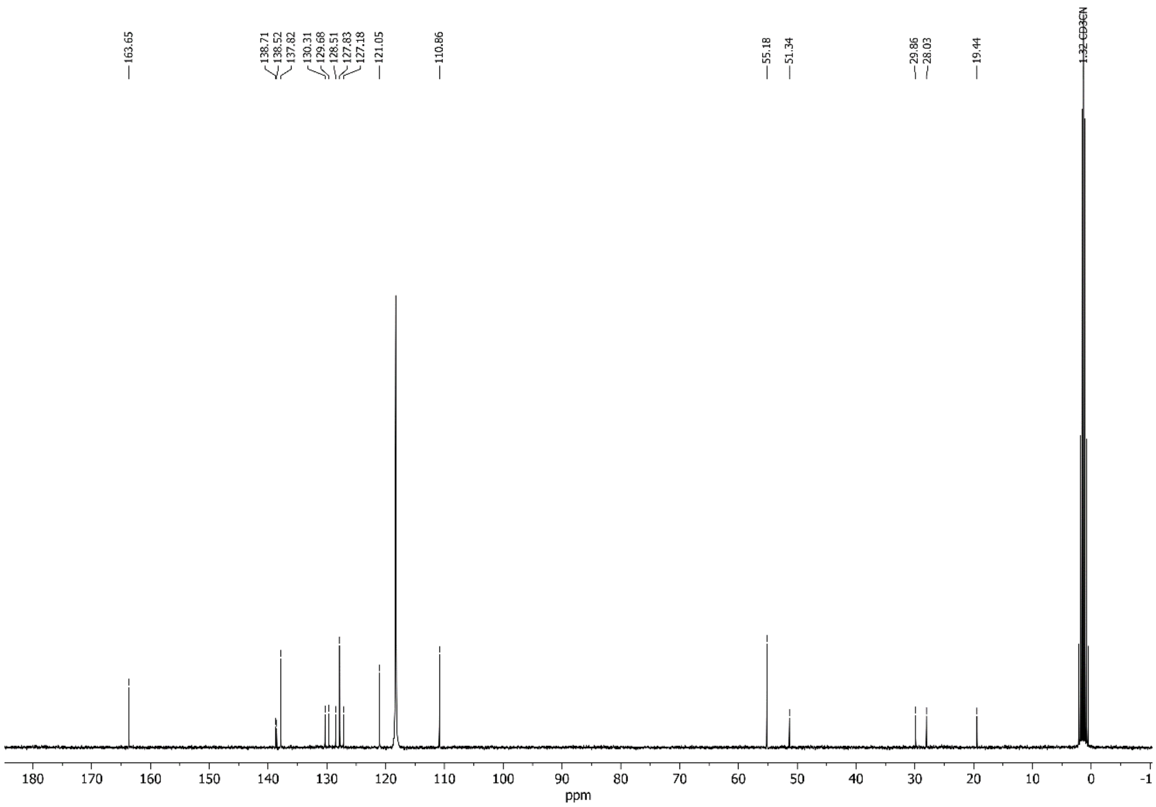

Fig. S11. 75 MHz ^{13}C-NMR of **2b** in ACN-*d3*

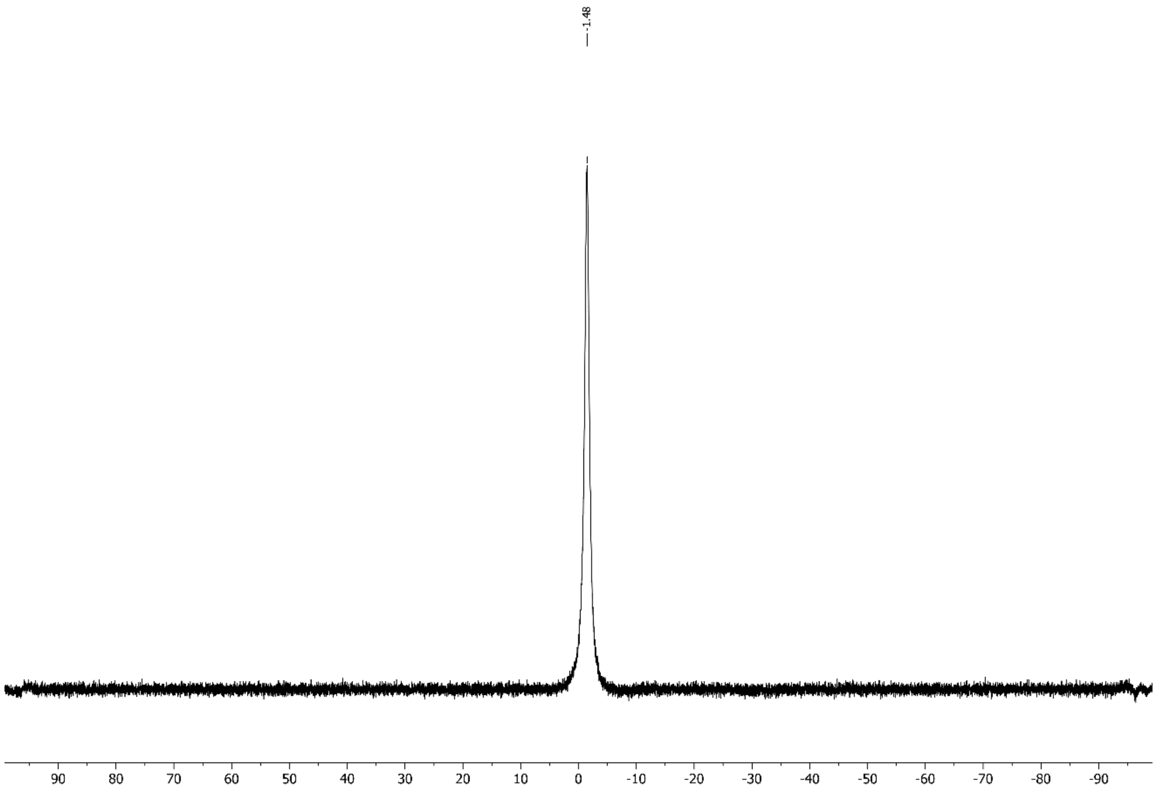

Fig. S12. 128 MHz ^{11}B-NMR of **2b** in ACN-*d3*

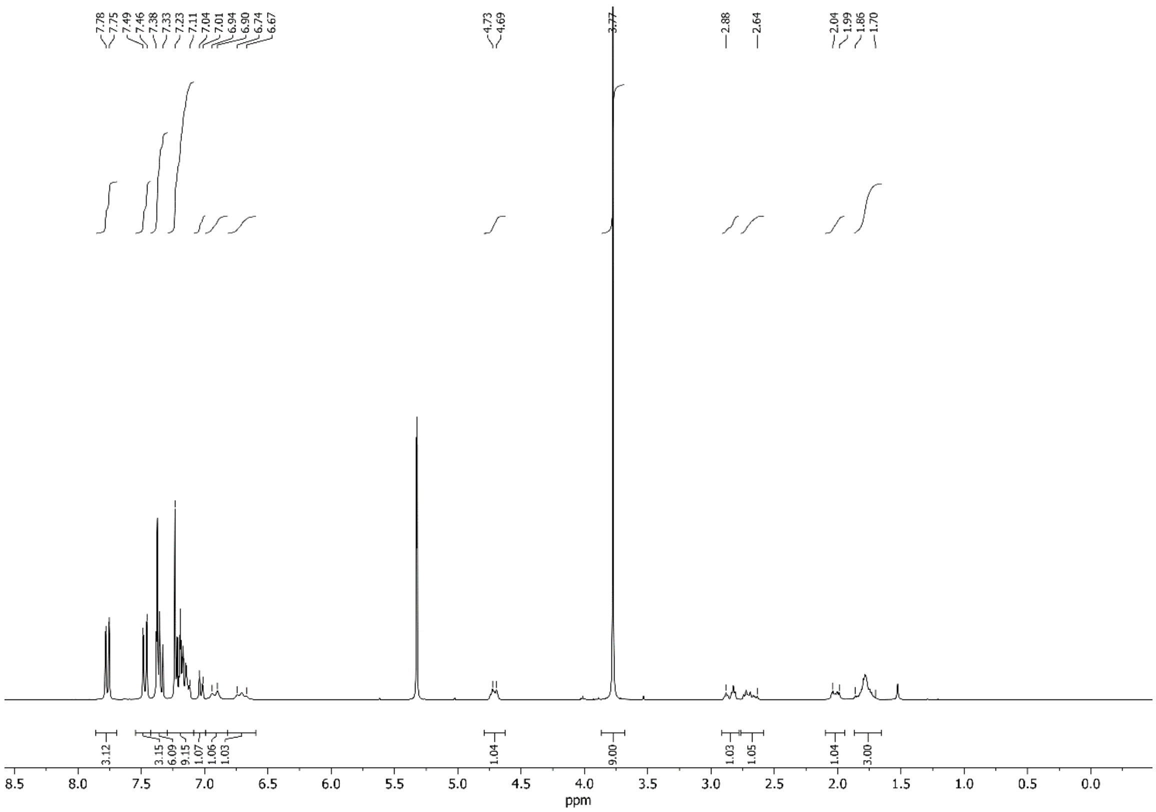

Fig. S13. 300 MHz ^{1}H-NMR of **2c** in DCM-*d2*

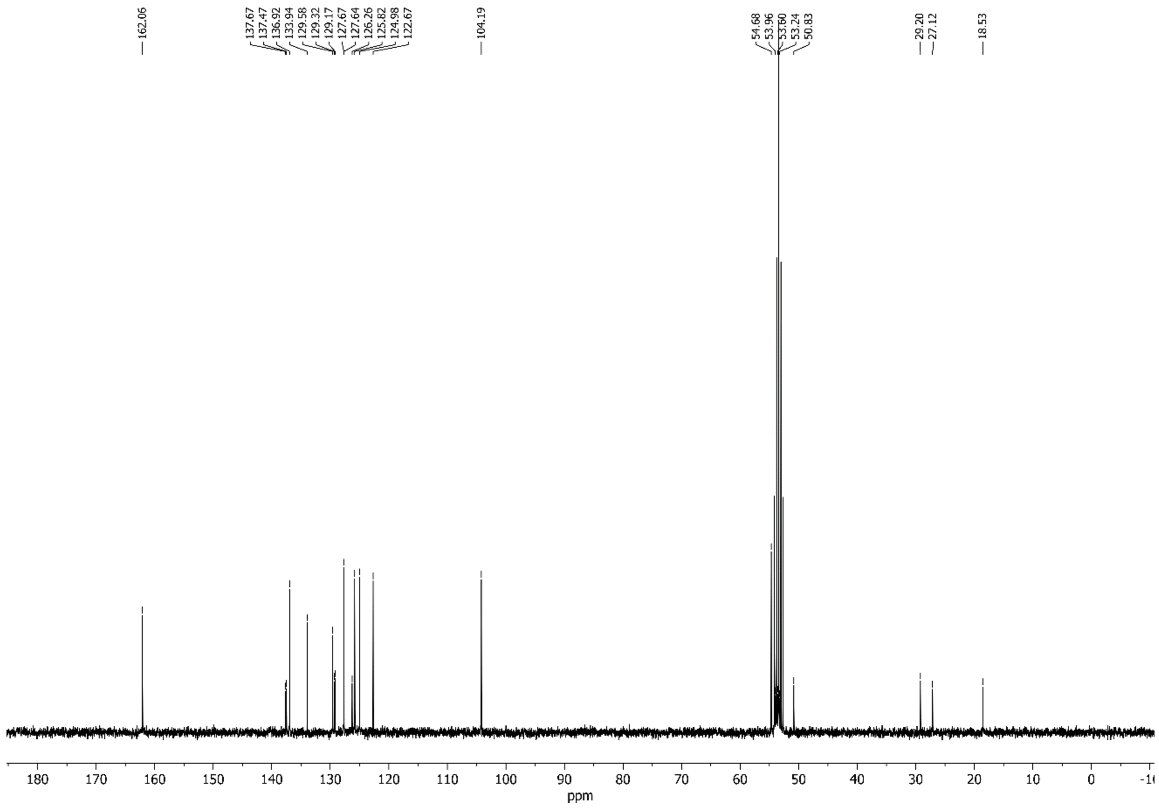

Fig. S14. 75 MHz ^{13}C-NMR of **2c** in DCM-*d2*

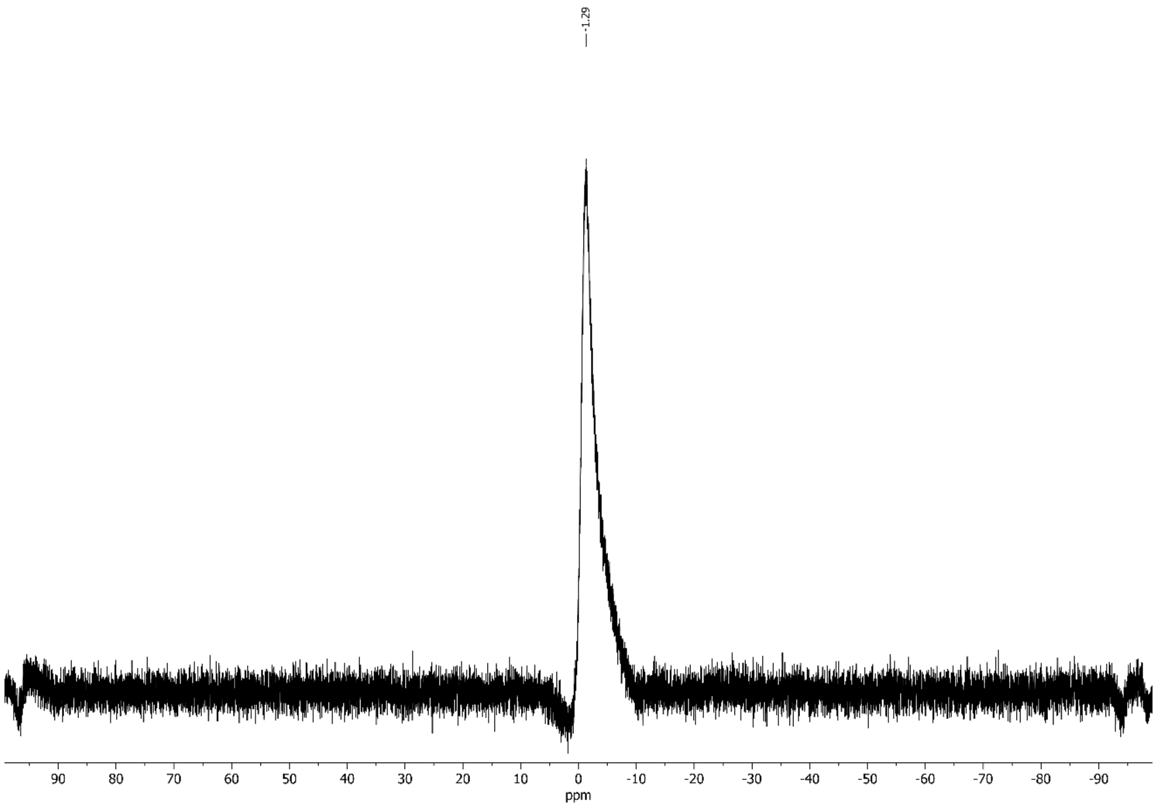

Fig. S15. 128 MHz ^{11}B-NMR of **2c** in DCM-*d2*

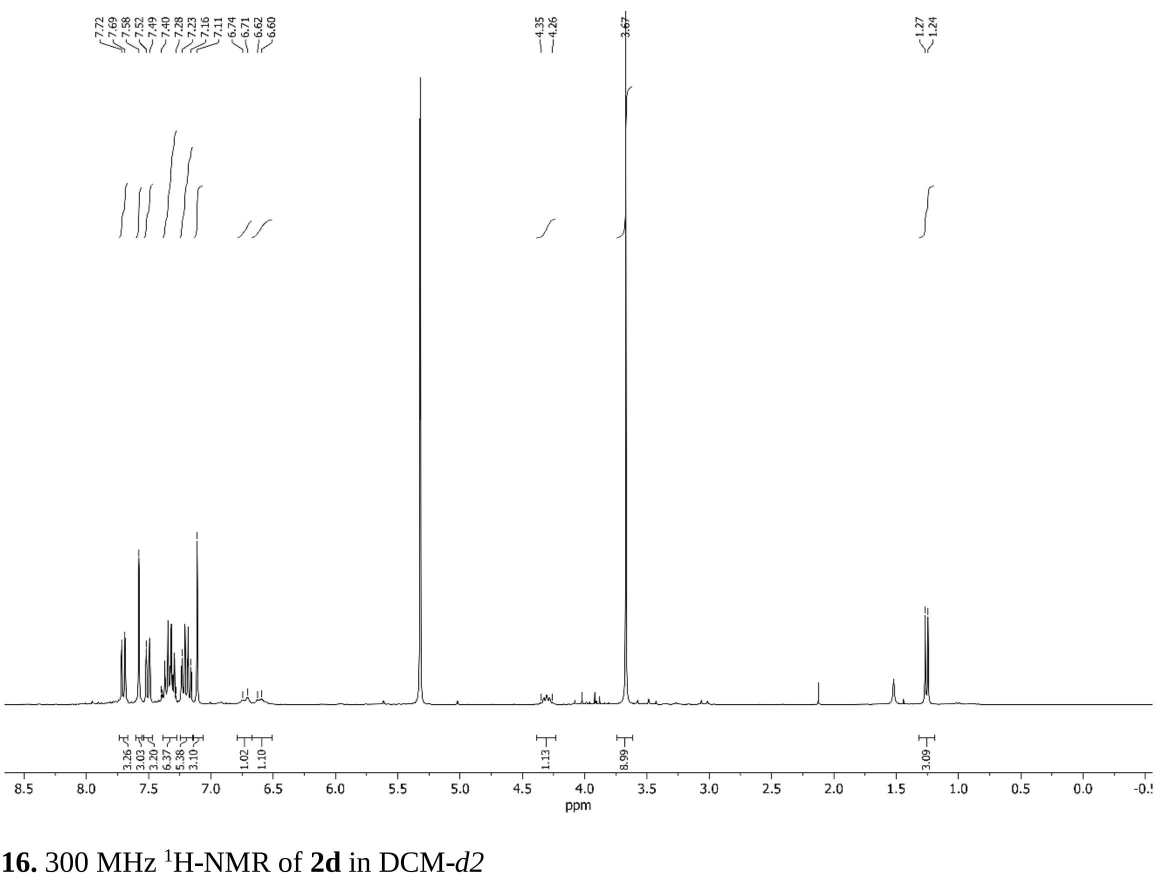

Fig. S16. 300 MHz ^{1}H-NMR of **2d** in DCM-*d2*

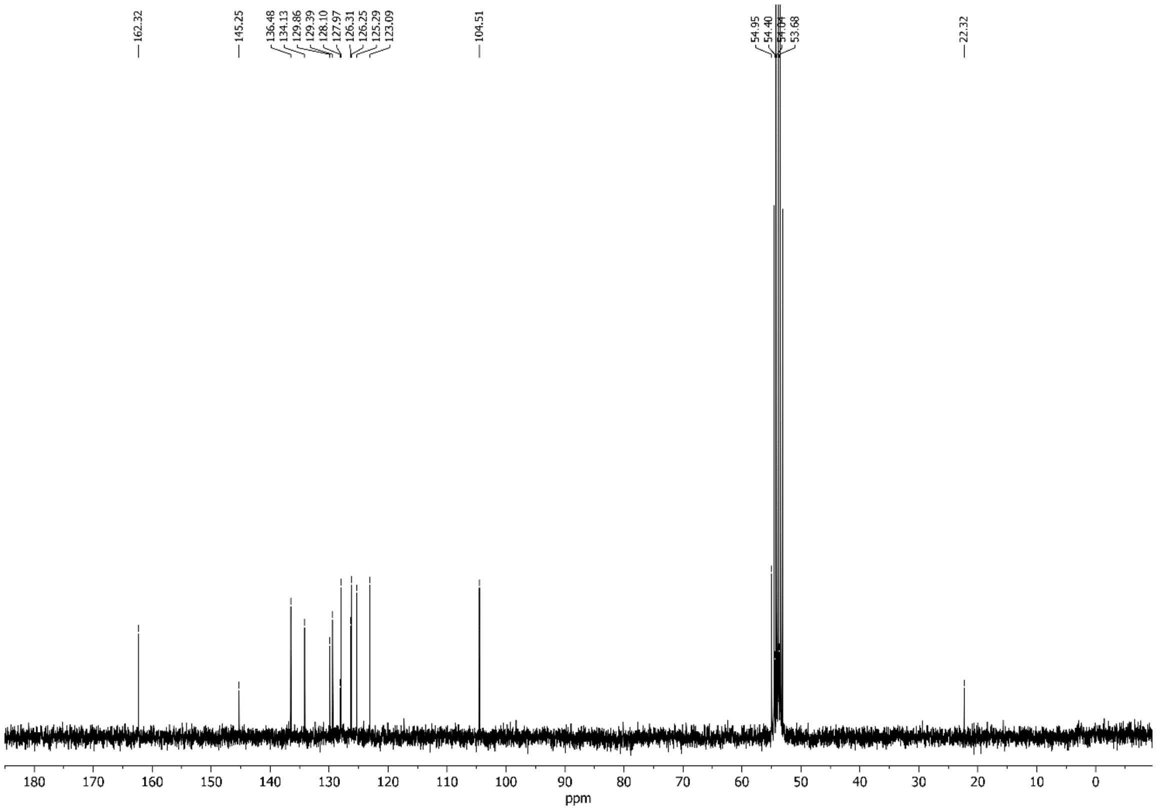

Fig. S17. 75 MHz ^{13}C-NMR of **2d** in DCM-*d2*

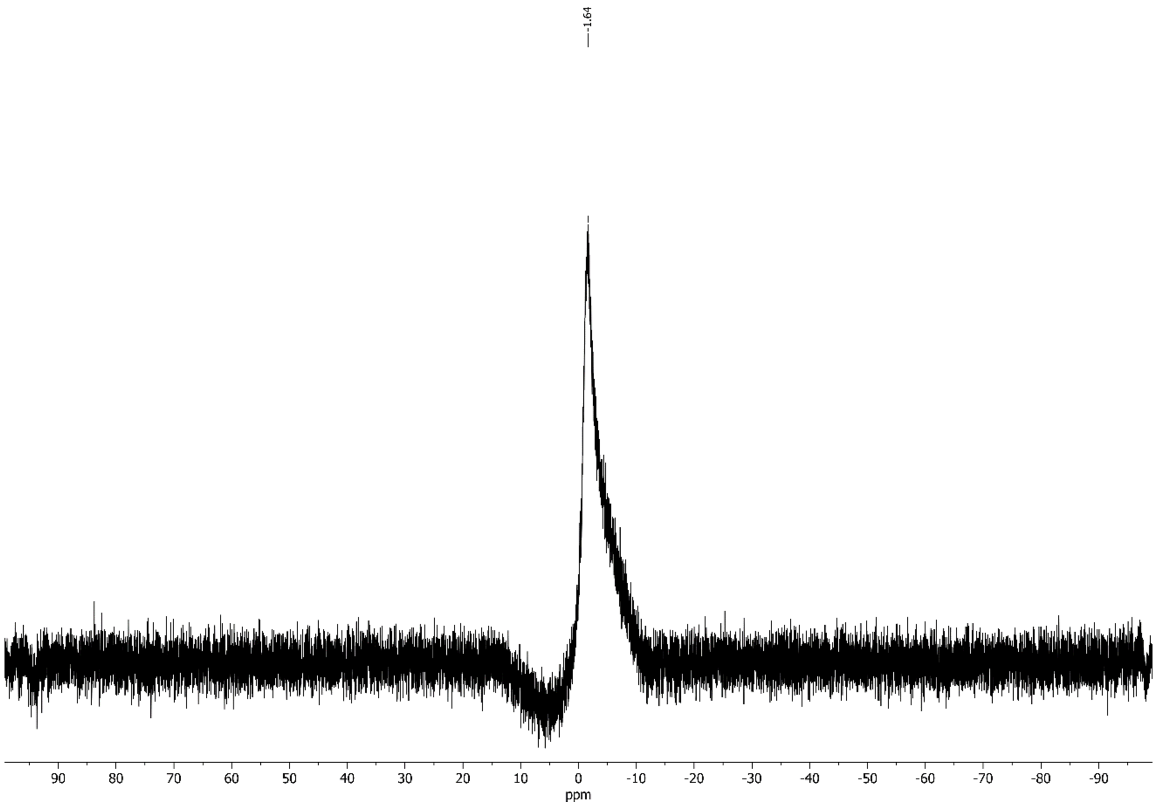

Fig. S18. 128 MHz ^{11}B-NMR of **2d** in DCM-*d2*

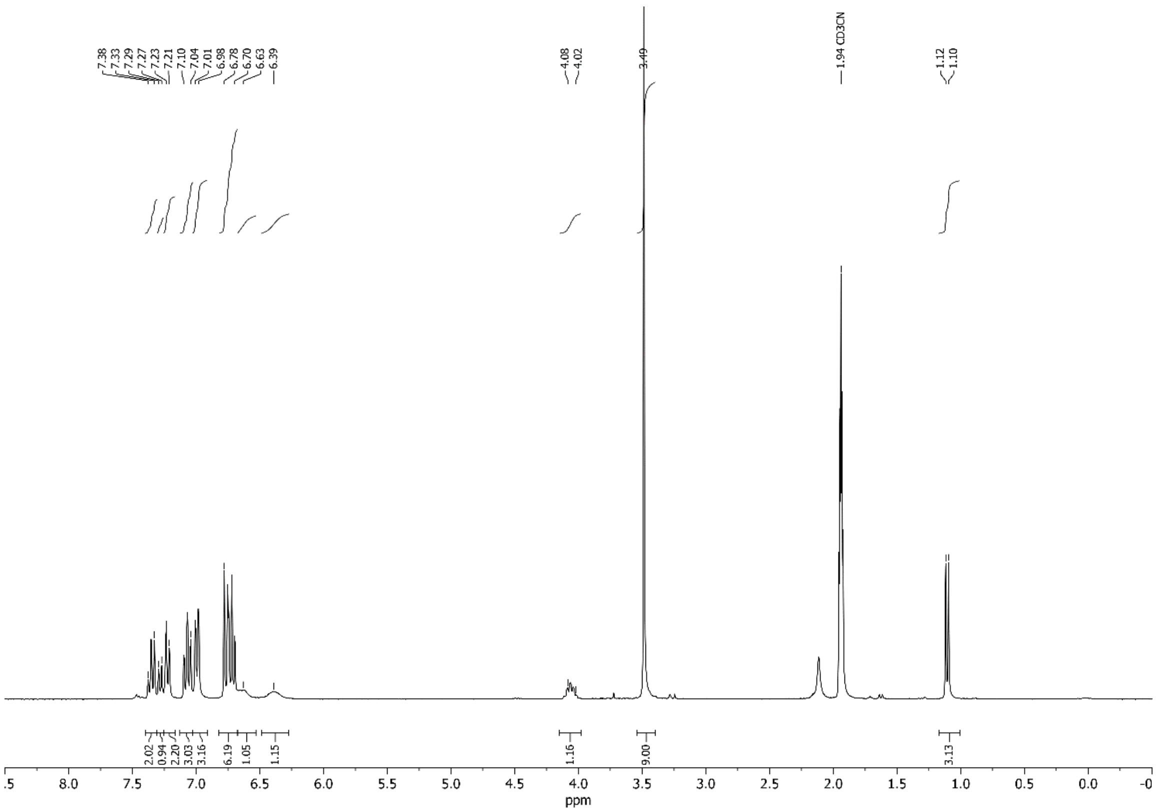

Fig. S19. 300 MHz ^{1}H-NMR of **2e** in ACN-*d3*

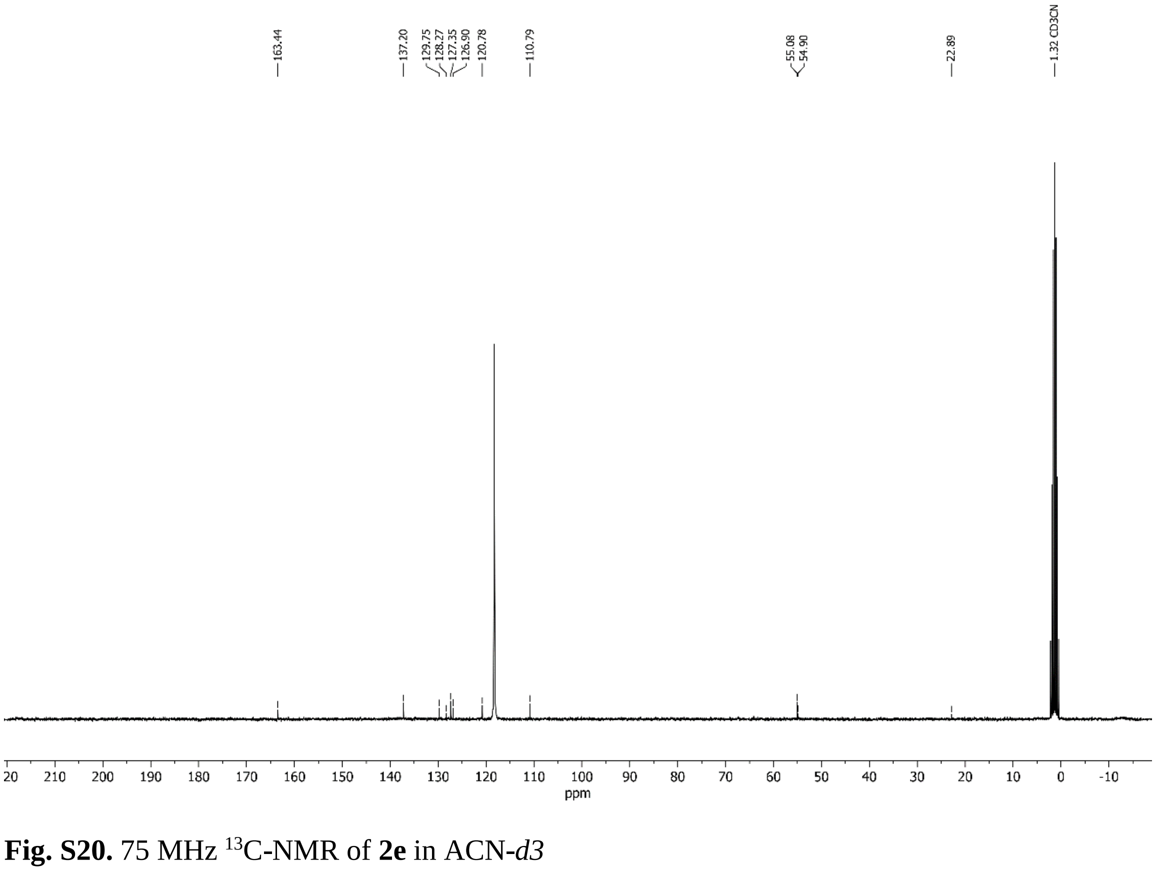

Fig. S20. 75 MHz ^{13}C-NMR of **2e** in ACN-*d3*

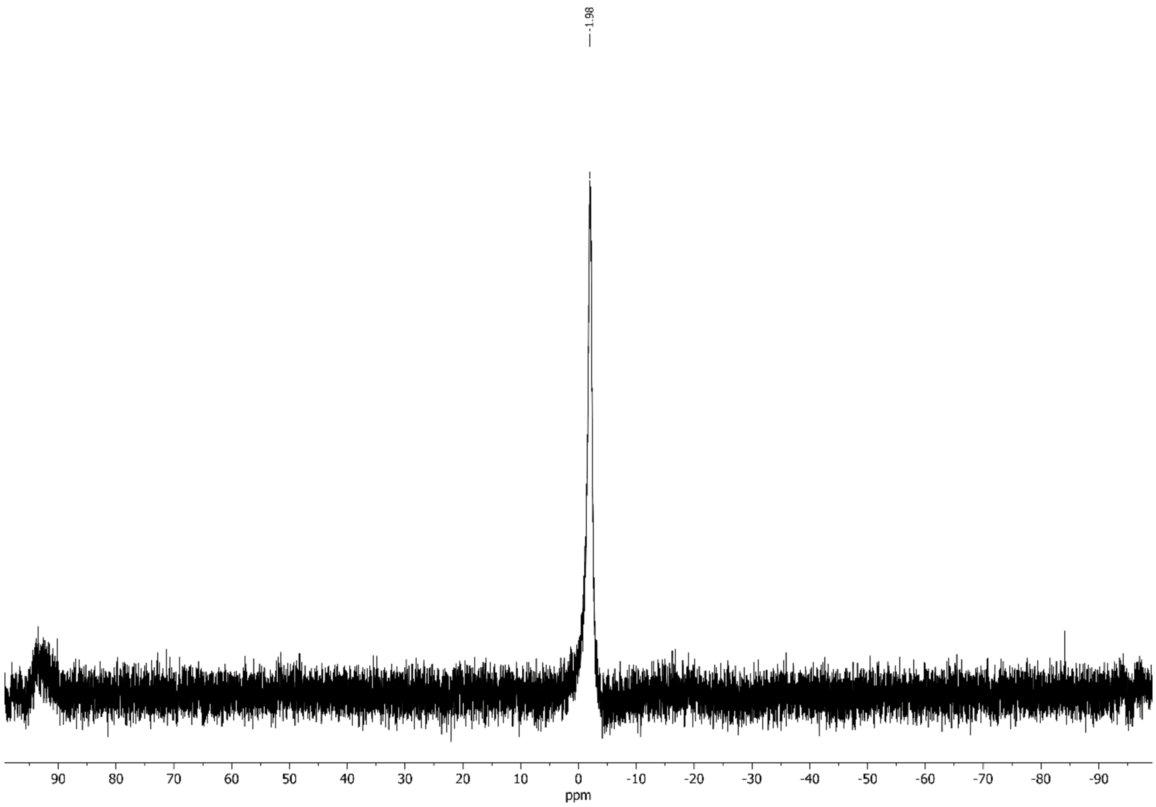

Fig. S21. 128 MHz ^{11}B-NMR of **2e** in ACN-*d3*

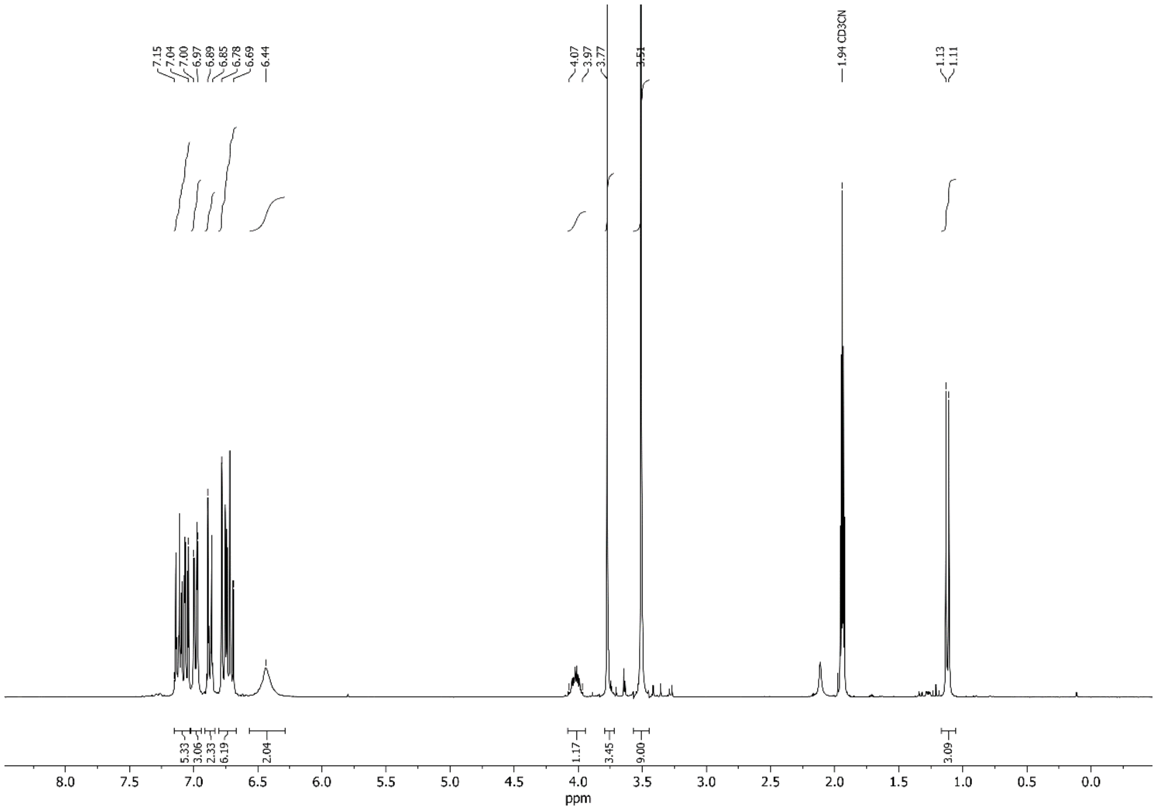

Fig. S22. 300 MHz ^{1}H-NMR of **2f** in ACN-*d3*

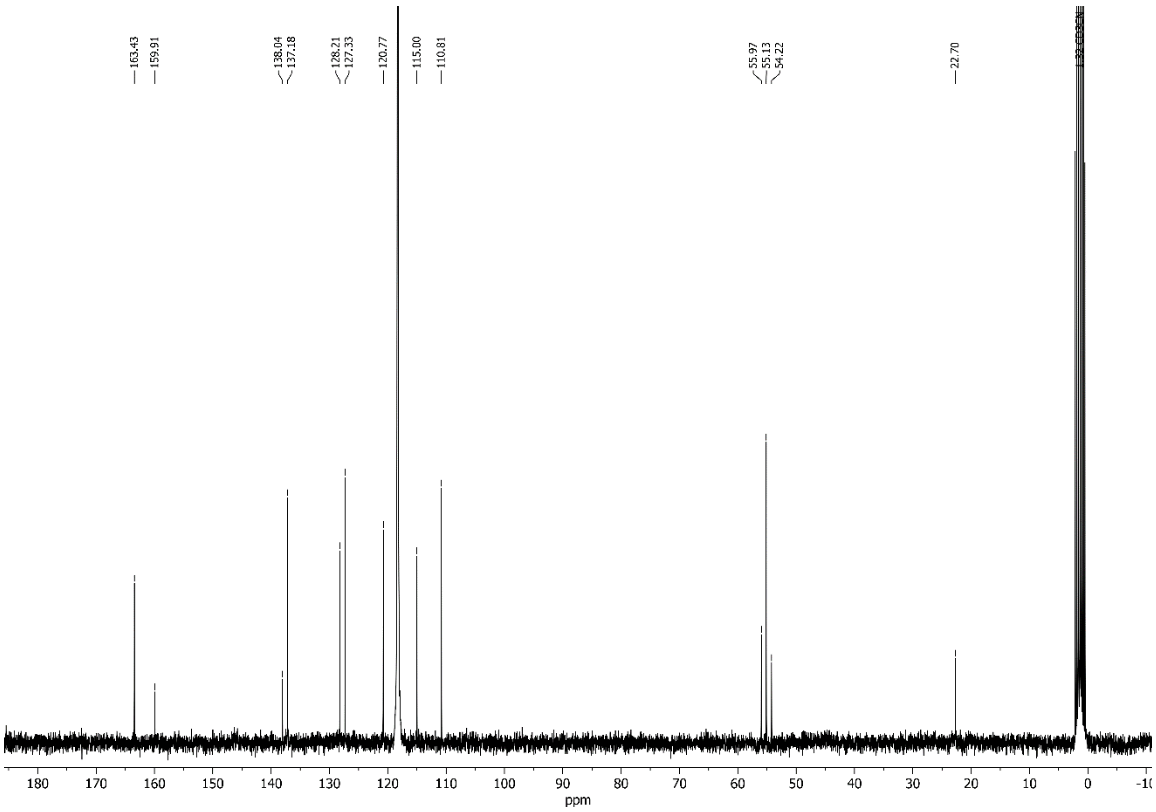

Fig. S23. 75 MHz ^{13}C-NMR of **2f** in ACN-*d3*

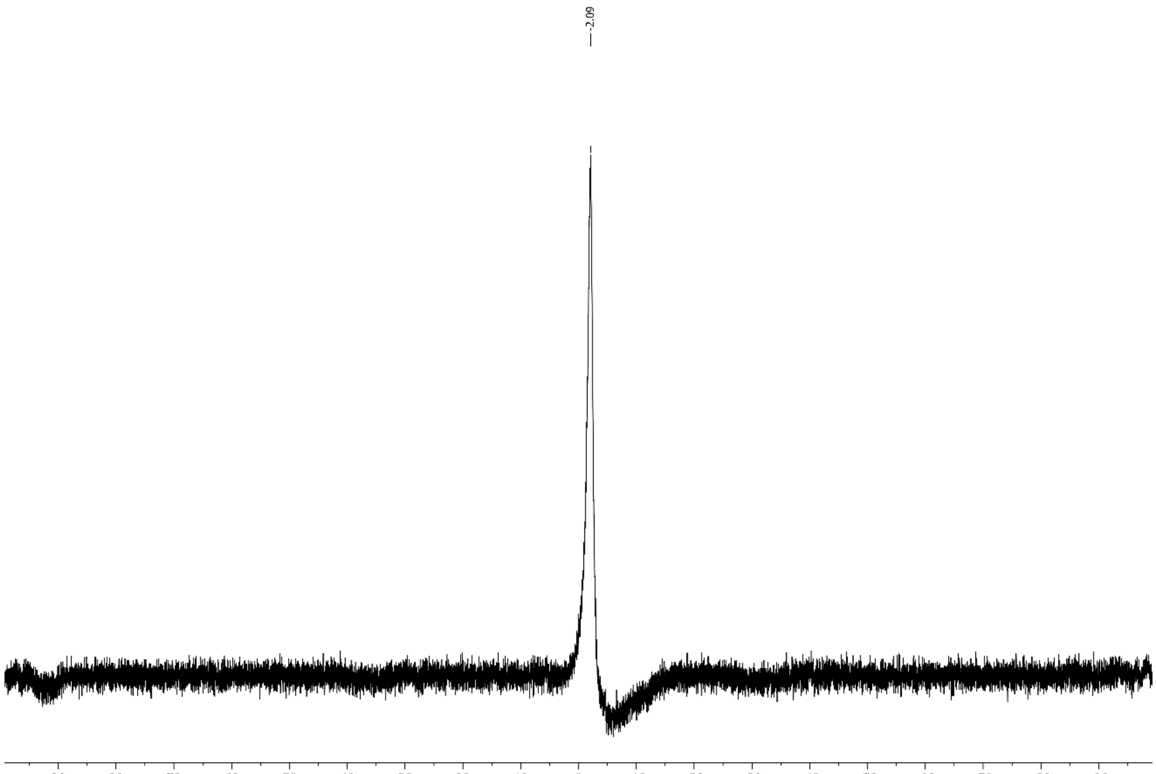

Fig. S24. 128 MHz ^{11}B-NMR of **2f** in ACN-*d3*

6. Selected Cartesian coordinates

For structures of MMM^{3u} and PPP^{3u} of **2a**, see crystal structure.

2b-PPP^{3u}

```
C -1.88932500    0.83797700   -0.66375300
C -1.78266300    2.20196400   -1.04426900
C -3.09806800    0.22093400   -1.02632400
C -2.80272500    2.87071100   -1.73306700
C -4.12954200    0.86460800   -1.71831100
H -3.25106000   -0.81454000   -0.74545900
C -3.97808700    2.19889700   -2.07379900
H -2.69289600    3.91184300   -2.00855300
H -5.03864900    0.32475800   -1.96543600
H -4.76206100    2.72737600   -2.60825200
C -1.31274500   -1.51936300    0.57789800
C -1.77427200   -1.80598800    1.87210000
C -1.41558000   -2.59154100   -0.34184400
C -2.29052500   -3.04787100    2.25466700
H -1.73149800   -1.01875900    2.61747200
C -1.91398400   -3.84864900    0.01605100
C -2.35381600   -4.07636700    1.32164600
H -2.63478400   -3.20364600    3.27256600
H -1.97089500   -4.65160500   -0.70778000
H -2.74319200   -5.05302400    1.59288600
C -0.30648600    0.76519200    1.59390700
C  0.79785200    0.36526200    2.38517900
C -1.08314500    1.79771400    2.13907900
C  1.11732000    0.98202200    3.59983400
C -0.79908800    2.42051100    3.35836600
H -1.95795700    2.12691100    1.58829700
C  0.31401900    2.01544300    4.08671200
H  1.97505400    0.66203800    4.17775800
H -1.44300900    3.21249300    3.72878200
H  0.56308200    2.48683800    5.03276900
O -1.00564700   -2.33011400   -1.63862500
O -0.62091700    2.84476800   -0.70287700
O  1.54644300   -0.69059100    1.90264200
C  2.61096700   -1.21109100    2.68822400
H  2.24780600   -1.56300500    3.66056100
H  3.01414100   -2.05394200    2.12515200
H  3.40134200   -0.46658500    2.83834900
C -1.17643200   -3.32861600   -2.63762000
H -2.23036600   -3.61229500   -2.73511100
H -0.83698300   -2.87913900   -3.57195700
H -0.57497800   -4.21991400   -2.42350800
C -0.49006900    4.23357800   -0.96185200
H  0.48327600    4.52205200   -0.56104500
H -0.51604700    4.45028200   -2.03728700
H -1.27323300    4.81198100   -0.45670700
B -0.76216400    0.00324600    0.20976000
C  1.45991400    0.75935200   -1.36258300
C  0.92480600    1.02276300   -2.79255500
C  1.66031300    0.26024100   -3.92327600
C  2.42625300   -0.97696600   -3.42102300
H  0.98846100    2.09696400   -2.98422900
H -0.14076000    0.78096900   -2.80441200
H  0.94516100   -0.02803500   -4.70060600
H  2.38769900    0.92761700   -4.39954700
H  1.72160200   -1.73380400   -3.04855500
H  2.97241500   -1.44869500   -4.24399700
H  1.33871200    1.65547200   -0.75897100
N  0.61955500   -0.30210500   -0.67997200
H  0.35110400   -1.01577700   -1.36024600
H  1.21369800   -0.76649200    0.01116200
C  2.91745900    0.33853500   -1.35243400
C  3.79762300    0.81581400   -0.37684400
C  3.38316500   -0.55665800   -2.33179100
C  5.12997300    0.40082500   -0.35441300
H  3.43549600    1.51597700    0.37078100
C  4.71862600   -0.96721700   -2.30318800
C  5.58936000   -0.49864400   -1.31747700
H  5.80458300    0.78038000    0.40645300
H  5.08220900   -1.65061900   -3.06584300
H  6.62466200   -0.82520600   -1.30994800
```

2b-MMM^{3u}

```
C -1.99350500    0.85783400   -0.84022800
C -2.26201100    0.42973300   -2.16179100
C -2.88515200    1.81349500   -0.33257000
C -3.31522300    0.94791600   -2.92262400
C -3.95542000    2.33858400   -1.06319200
H -2.74252400    2.15761800    0.68655100
C -4.16491700    1.90832500   -2.36884800
H -3.48913900    0.61012300   -3.93640400
H -4.61431900    3.07450300   -0.61221900
H -4.98408400    2.30412800   -2.96171200
C -1.29676100   -1.36214900    0.50069400
C -2.64976800   -1.73056900    0.42998600
C -0.42787000   -2.38332400    0.95986200
C -3.12861300   -2.99960800    0.76993300
H -3.36683900   -0.98838100    0.09585600
C -0.87581300   -3.66628900    1.29141500
C -2.23419200   -3.97457000    1.19636200
H -4.18974200   -3.21770800    0.69671900
H -0.18348600   -4.42621500    1.63068000
H -2.57738000   -4.97056500    1.45953000
C -0.50800200    1.06401800    1.47739400
C -0.22018800    2.45451100    1.49911300
C -0.55291300    0.45297000    2.74158800
C  0.01395800    3.15327500    2.69111100
C -0.32223900    1.12754900    3.94477300
H -0.79142500   -0.60292700    2.79389100
C -0.03472400    2.48677200    3.91638800
H  0.23211500    4.21353000    2.67627700
H -0.37437000    0.59188100    4.88779800
H  0.14738400    3.03827400    4.83412100
O  0.90761800   -2.04178300    1.08246000
O -1.42491500   -0.54683100   -2.67137200
O -0.18558200    3.09078700    0.28500100
C -0.01243500    4.49840800    0.24247900
H  0.96805200    4.79925300    0.63309100
H -0.07972700    4.77568800   -0.81072300
H -0.79972400    5.01701700    0.80308800
C  1.84188100   -3.00903700    1.54717800
H  1.88725000   -3.87657800    0.87857300
H  2.81024200   -2.50711600    1.55538000
H  1.59472000   -3.34002900    2.56218100
C -1.73709000   -1.14270100   -3.92446900
H -1.00185600   -1.93521600   -4.07259600
H -2.74205200   -1.57939000   -3.91390800
H -1.66035500   -0.41980200   -4.74541100
B -0.83798400    0.19069200    0.11681600
C  1.57795000    1.04848300   -1.09051100
```

C	1.13421200	1.90832500	-2.29214400
C	1.58312100	1.38780600	-3.68618000
C	2.19166600	-0.02824300	-3.66155900
H	1.55002000	2.90750300	-2.13639200
H	0.05033400	2.02463200	-2.24759700
H	0.73955600	1.40994900	-4.38326200
H	2.34020200	2.06652200	-4.09415800
H	1.40020600	-0.77260100	-3.49970100
H	2.63222200	-0.26600400	-4.63502800
H	1.62392000	1.67429000	-0.20266000
N	0.56482500	-0.02941300	-0.76885300
H	0.24509600	-0.46533700	-1.63628100
H	1.05687900	-0.75653700	-0.24294400
C	2.93771200	0.41553500	-1.31691300
C	3.89269300	0.36999400	-0.29701700
C	3.23411100	-0.13633300	-2.57551500
C	5.13566200	-0.22816100	-0.51169800
H	3.66179100	0.80746200	0.67063200
C	4.48258000	-0.72854500	-2.78420800
C	5.42909400	-0.78113400	-1.75894500
H	5.87065900	-0.25549700	0.28645400
H	4.71796500	-1.14642200	-3.75935300
H	6.39488100	-1.24376200	-1.93722500

2c-PPP^{3u}

C	4.55506000	3.44683000	3.39776800
C	5.29744000	3.74118600	4.52465200
C	5.48874100	5.05254100	5.04002800
C	4.87710400	6.14714600	4.36047500
C	4.12238100	5.87705100	3.18910300
C	3.98274600	4.58422700	2.72622400
H	6.72605000	4.47057900	6.71575700
H	5.77874500	2.92975700	5.06192600
C	6.25952600	5.30837000	6.20428000
C	5.05390700	7.45993400	4.87259900
H	3.67375900	6.71272100	2.66586600
C	5.80620800	7.67792200	6.00752400
C	6.41687900	6.59249800	6.68145600
H	4.58825400	8.29413300	4.35457600
H	5.93429400	8.68685000	6.38783600
H	7.00879900	6.77584000	7.57272800
O	3.29220900	4.30074200	1.56988200
C	4.96518400	0.77417300	3.90787400
C	5.98477200	-0.10419200	3.58757400
C	4.45657700	0.64892400	5.25303900
C	6.51513300	-1.08668200	4.46913900
H	6.43381300	-0.05120400	2.60182300
C	4.93766600	-0.28612700	6.14972600
C	7.55966200	-1.96741700	4.08600200
C	5.97515600	-1.18196800	5.78415600
H	4.53557000	-0.35533600	7.15307400
C	8.04972100	-2.90929900	4.96632400
H	7.97003600	-1.88612600	3.08290800
C	6.49658800	-2.15982000	6.67238900
C	7.51051500	-3.00468000	6.27208600
H	8.84944400	-3.57774000	4.66309900
H	6.08553500	-2.23280300	7.67579000
H	7.90093000	-3.74753500	6.96115700
C	5.38395400	1.81302600	1.42324400
C	6.37010200	2.74692700	1.16600400
C	5.26517900	0.76596800	0.44185900
C	7.22154800	2.72426900	0.02731600
H	6.52105500	3.55645000	1.87357000
C	6.05681300	0.69943800	-0.68635600
C	8.22160500	3.70701700	-0.19404800
C	7.05713300	1.67728600	-0.92620200
H	5.93950800	-0.09897800	-1.40880200
C	9.02574800	3.65834500	-1.31293300
H	8.34316100	4.50201100	0.53680500
C	7.89758100	1.64956700	-2.07062500
C	8.86019700	2.61844300	-2.25979800
H	9.78764000	4.41505500	-1.47170100
H	7.77195600	0.85133600	-2.79729100
H	9.49715200	2.58589500	-3.13852000
O	3.46331100	1.52434100	5.59192100
O	4.30554800	-0.18900500	0.70880000
C	4.18011200	-1.30891300	-0.16152600
H	3.88165600	-1.00010900	-1.17004600
H	5.11843300	-1.87213000	-0.21242500
H	3.40254300	-1.93935500	0.27164800
C	2.77426500	5.37038400	0.78736700
H	3.57627100	6.04561100	0.46892600
H	2.31788600	4.90739200	-0.08826500
H	2.01320700	5.93514900	1.33768800
C	2.95365700	1.51634600	6.91719100
H	2.47797900	0.55798300	7.16018100
H	3.74284700	1.72577500	7.64916500
H	2.20672000	2.31086400	6.95536700
B	4.47988200	1.91250300	2.81342000
C	1.69957400	1.51366600	3.15546200
C	1.51940500	0.03952500	3.59948300
C	0.49145000	-0.77534600	2.77685100
C	0.24844100	-0.19540400	1.37231500
H	1.22108500	0.03538400	4.65080800
H	2.49746100	-0.44724300	3.56591400
H	0.81965700	-1.81745000	2.70575000
H	-0.47079700	-0.78509100	3.30159600
H	1.16354800	-0.27002700	0.76773000
H	-0.51333800	-0.77724200	0.84400400
H	1.90635000	2.13308900	4.02459500
N	2.92421700	1.64229800	2.26857500
H	2.98972600	0.82272600	1.66191100
H	2.77419100	2.44733900	1.65577400
C	0.48849000	2.06116000	2.42377200
C	0.05253600	3.37222500	2.63786800
C	-0.19164600	1.24269000	1.50457800
C	-1.04029100	3.88344400	1.93648900
H	0.57390800	3.99680100	3.35794700
C	-1.28512000	1.76190200	0.80623400
C	-1.70641700	3.07678300	1.01287800
H	-1.37134200	4.90181700	2.11344500
H	-1.81827700	1.12717200	0.10364600
H	-2.55921200	3.46453100	0.46434800

2c-MMM^{3u}

C	5.20637700	0.79607900	2.92479300
C	6.21095600	0.31563300	2.10805000
C	6.60684400	-1.04839700	2.04157200
C	5.93119800	-1.99433300	2.86794100
C	4.88085700	-1.53157100	3.70340500
C	4.53070700	-0.19689000	3.71637000
H	8.15759100	-0.77449800	0.55904800
H	6.74024200	1.01166500	1.46418100
C	7.64894500	-1.49902000	1.18950800
C	6.32612400	-3.35749500	2.81813200
H	4.36080500	-2.25599500	4.31821000
C	7.34461400	-3.76574800	1.98289300
C	8.01326700	-2.82861100	1.15838900
H	5.81149700	-4.07788700	3.44845600
H	7.63629000	-4.81122300	1.95383900
H	8.81194400	-3.16122300	0.50275700
O	3.49147300	0.27162300	4.49146300
C	3.27330900	2.43611400	2.04888500
C	2.89480300	1.40510300	1.20877000

C	2.34595500	3.53699800	2.11251800
C	1.68851500	1.38036000	0.45624500
H	3.55306700	0.54855600	1.10245700
C	1.15483500	3.56062800	1.41636700
C	1.34544900	0.29673400	-0.39414300
C	0.79141000	2.48244100	0.56856300
H	0.47736800	4.40230500	1.49230200
C	0.16270700	0.30071100	-1.10267200
H	2.03374700	-0.54010500	-0.47703900
C	-0.42091400	2.46249800	-0.17086000
C	-0.72834100	1.39544400	-0.98814100
H	-0.08925300	-0.53332700	-1.75013200
H	-1.10447000	3.30293600	-0.08469900
H	-1.65791000	1.39210500	-1.54922500
C	5.84274900	3.35748900	2.12726500
C	5.47424000	4.16015000	1.06214300
C	7.23820100	3.43075400	2.48995500
C	6.35672400	5.02361100	0.35612200
H	4.44614100	4.13711400	0.71742800
C	8.13708900	4.25347800	1.83704200
C	5.92499200	5.83021200	-0.72884300
C	7.72331400	5.07425800	0.75690200
H	9.17859400	4.28729200	2.13257000
C	6.80744400	6.65861800	-1.38967500
H	4.88244300	5.78317700	-1.03225400
C	8.61274600	5.93516200	0.06021600
C	8.16461800	6.71018100	-0.98860100
H	6.46667500	7.27112400	-2.21853800
H	9.65517100	5.97398900	0.36507600
H	8.85491700	7.36377200	-1.51336300
O	2.73288900	4.58971600	2.91504000
O	7.62117300	2.62429900	3.52525900
C	8.99267900	2.56961800	3.88919800
H	9.61559900	2.24496900	3.04715400
H	9.35237800	3.54012600	4.25346400
H	9.05943800	1.83435000	4.69225900
C	2.67860400	-0.66255700	5.19348600
H	3.25065300	-1.18938500	5.96613900
H	1.88609700	-0.07874100	5.66357000
H	2.23484600	-1.39005100	4.50497700
C	1.88639400	5.72863700	3.03419100
H	1.74449500	6.21420800	2.06239400
H	0.91191100	5.45762200	3.45631200
H	2.40016800	6.41147100	3.71163300
B	4.73466000	2.36585800	2.84384000
C	5.38459100	3.38144700	5.40899900
C	6.00210900	2.18697200	6.16362700
C	5.22198300	1.71987100	7.42470700
C	3.84897500	2.39834100	7.60423400
H	7.00887200	2.49138500	6.46253900
H	6.13541300	1.36560400	5.45777600
H	5.09004600	0.63362500	7.40287400
H	5.82035500	1.94086200	8.31518100
H	3.13211600	1.98948600	6.87922100
H	3.44067100	2.16431000	8.59245200
H	6.16457900	3.88893400	4.84716100
N	4.37347000	2.92973500	4.37558600
H	3.78192000	2.19957200	4.77744800
H	3.76059700	3.72760900	4.18822300
C	4.73689900	4.37389400	6.35540900
C	4.89658700	5.75133500	6.17818000
C	3.96468600	3.89260800	7.42685100
C	4.28729100	6.65618200	7.04918900
H	5.50378700	6.11726100	5.35452900
C	3.36231000	4.80469500	8.29759500
C	3.51597200	6.17985200	8.11024200
H	4.42010500	7.72353000	6.90404900
H	2.77294600	4.43506800	9.13238800
H	3.04260400	6.87563100	8.79603000

7. References

3. Meyer, M., Paciorek, W., Kowalski, A., Muszynski, A., Wisniewski, A., Pol, M., Przewozniczek, M., Stec, P., Bujnik, D., Kulza, H., Grzesczyk, M., Kuna, T., Serwata, L., Prochniak, G., Piotrowski, W., Antkowiak, M., Jasiak, R., Idzi, M., Yoshida, H., Hendrixson, T., Archeson, C. & Wodziczko, T. (2018). CrysAlisPro: Rigaku Oxford Diffraction (1995-2018).

4. Farrugia, L. J. WinGX suite for small-molecule single-crystal crystallography. J. Appl. Crystallogr. 1999, 32, 837–838, DOI: 10.1107/S0021889899006020.

5. Sheldrick, G. M. A short history of SHELX. Acta Crystallogr A Found Crystallogr 2008, 64, 112–122, DOI: 10.1107/S0108767307043930.

6. Hübschle, C. B.; Sheldrick, G. M.; Dittrich, B. ShelXle: a Qt graphical user interface for SHELXL. J. Appl. Crystallogr. 2011, 44, 1281–1284, DOI: 10.1107/S0021889811043202. 5 Putz, H.; Brandenburg, K. Diamond; Crystal Impact: Bonn, Deutschland, 2014
